# Molecular Dynamics Study of the Coalescence of Water Droplets with Anionic Asphaltene Molecules under a DC Electric Field

**DOI:** 10.1021/acs.langmuir.5c02957

**Published:** 2025-09-02

**Authors:** Jurgen Lange Bregado, Argimiro R. Secchi, Marcio Nele

**Affiliations:** † Chemical Engineering Program, COPPE, 28125 Universidade Federal do Rio de Janeiro, Cidade Universitária, Rio de Janeiro, CP 21941-914, Brazil; ‡ Chemical and Biochemical Process Engineering Program, Escola de Química, Universidade Federal do Rio de Janeiro, Cidade Universitária, Rio de Janeiro, CP 21941-909, Brazil

## Abstract

During the extraction of crude oil, water-in-oil (W/O) emulsions are mostly formed at a high pH, where water droplets can be stabilized by anionic asphaltene molecules on the surface. The study of driving forces in the electro-coalescence of these emulsions is fundamental to the efficient design of the oil dehydration process. We studied by molecular dynamics the electro-coalescence of two asphaltene-laden droplets suspended in *n*-hexane as a model oil. The findings indicate that a low number of anionic asphaltenes per water droplet and a moderate electric field strength (*E*) allow optimal droplet–droplet coalescence conditions to be reached, which is favored by high electrical polarization of water droplets. Under these conditions, it has been found that the diffusion and polarity of water molecules are enhanced, favoring the formation of the liquid bridge between colliding droplets and reducing the droplet–droplet coalescence time. On the contrary, with a high number of asphaltenes per droplet and *E*, the droplet–droplet coalescence is hindered and/or retarded due to the steric effect of asphaltene aggregation at the interface between water droplets. Here, the high ionic conductivity (σ) of water droplets and low interfacial tension (γ) before the formation of the liquid bridge led to the formation of a water chain (WC) between electrodes, an undesirable phenomenon impairing the dehydration efficiency in the coalescer. This study demonstrates that W/O emulsions with anionic asphaltenes under conditions of relatively low σ and somewhat high γ at moderate *E* (around the critical *E*) promote complete droplet–droplet coalescence and prevent WC formation.

## Graphical abstract

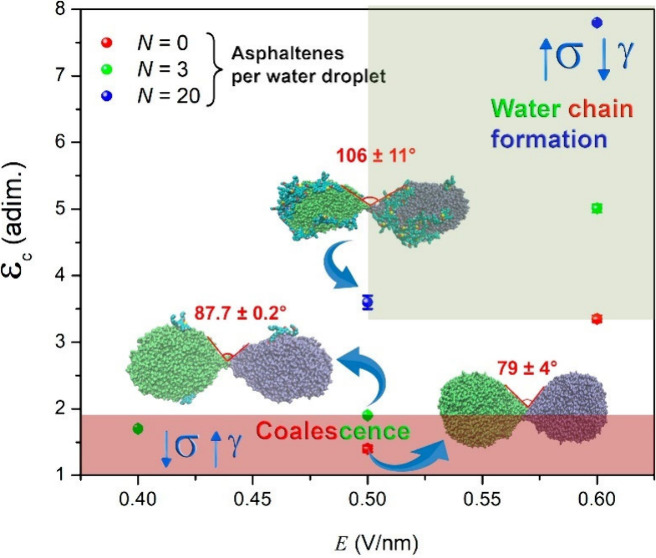

## 1 Introduction

Water droplets dispersed in crude oil are undesirable in the oil industry, leading to process equipment corrosion and possible catalyst poisoning.^1–4^ Furthermore, the cost of transporting water by pipeline or tanker, the use of chemicals, and the extra processing equipment required to produce quality crude oil add to the production cost.^1,5^ Therefore, several commercial and industrial reasons exist to remove this emulsified water from crude oil. Water droplets can be removed from a continuous oil phase by several methods, such as chemical demulsification,^6^ gravity or centrifugal settling,^7^ pH adjustment and heating treatment,^8^ membrane filtration separation,^9^ and electro-coalescence.^1,10–13^ The latter has been the standard method in crude oil desalting and dewatering where the electric field is applied to enhance the coalescence of aqueous droplets in crude oil, thus improving phase separation.^14–16^ Compared with other techniques, the electro-coalescence reduces the sedimentation time, the heat, and the chemical demulsifier used, which is environmentally friendly.^17^

Among the factors that affect the electro-coalescence process is the presence of surface active molecules such as asphaltenes, which consist of aromatic rings condensed and attached to aliphatic branches.^18,19^ They are the heaviest fraction of oil and are considered the most enigmatic component of petroleum due to their influence on stabilizing water-in-crude oil (W/O) emulsions, closely related to their self-aggregation.^20^ Generally, asphaltene hierarchical aggregation contributes to W/O emulsion stability by forming a network “gel-like” or “glassy” structure within a thin oil film, separating approaching water droplets and decreasing their interfacial tension.^21–23^ Strong interfaces created by asphaltene aggregation oppose film drainage and thin film breakage, making coalescence between droplets difficult.^16^ For the case of uncharged asphaltene molecules formed at neutral water pH, the increased self-aggregation capacity impairing droplet coalescence in W/O emulsions seems to be predictable with an increasing asphaltene concentration due to the greater number of aromatic nuclei that can aggregate by π–π stacking.^19^ Nevertheless, for anionic asphaltenes formed at high water pH,^24,25^ it is not clear how their concentration on the water–oil interface would affect emulsion stability under the application of an electric field. On one hand, at high asphaltene concentrations, the aggregation phenomenon of asphaltenes on droplet surfaces could be even more accentuated when there are charged groups in asphaltenes, such as carboxylate groups in aliphatic branches,^26^ due to its high surface activity (as a surfactant) at the oil/water interface.^24,27–29^ For example, applying an electric field might lead to the electrodeposition of asphaltenes at the water interface, thus contributing to the stabilization of emulsions.^30^ However, such surface activity might also increase the polarization of water droplet surfaces by applying an electric field, thus producing the opposite effect of stabilizing the emulsion with enhanced droplet coalescence. In other words, asphaltene-laden droplets held under electrical potentials can acquire a net surface charge of opposite polarity, leading to a Coulombic attraction on the adjacent interfaces of the pair of droplets.^16^ Indeed, experimental and molecular dynamics (MD) studies show that a droplet pair better coalesces, at a lower potential, when the droplet–oil interface is populated with surface active molecules such as asphaltenes and surfactants.^16,31^ It appears that the coalescence (and noncoalescence) process depends mainly on two competing phenomena: the electrical polarization of the water droplets and the steric self-aggregation of asphaltenes, which in turn depend on the concentration of asphaltenes. In this scenario of interfacial rearrangement, the orientation of asphaltenes under electric forces contributes to the droplet–droplet interaction, a fact that has not yet been clarified.^16^ To the best of our knowledge, until now, the behavior of carboxylate asphaltenes in W/O emulsions has not been studied in the electric field during the electro-coalescence process. The electro-coalescence literature lacks an understanding of how electrostatics and an interface populated with anionic asphaltenes influence each other as two water droplets approach and merge.

Some investigations have significantly deepened the understanding of an emulsion’s stabilization and demulsification mechanism.^1–13,18,22,24,27,28,32^ However, it is still a challenge to understand the aggregation of asphaltene by experimental methods since this behavior cannot be explained through the standard colloidal interaction models and the mesoscale aggregation theories.^26,33^ On the other hand, the theoretical study of the aggregation phenomenon is challenging because of the multitude of engaged interactions as hydrogen bonds, charge transfer, and π–π interactions by the presence of polar (N, O, and S) and nonpolar (C and H in aromatic rings) atoms composing the asphaltene molecules.^18,34–39^ In this complex picture of interactions, MD simulation is a valuable tool for experimental research that can allow us to understand these interactions at the molecular level. Through MD simulations, the dynamic evolution and molecular behavior at the oil/water interface can be captured and thus reveal the nature of asphaltene aggregation.^19,20,26,33,34,40,41^ Under the influence of an electric field, the dominant forces can be understood (electrostatic, van der Waals, hydrodynamics, etc.) as well as the underlying mechanism of coalescence of freely moving water droplets containing asphaltene molecules in the ionic state.^42,43^

The current work uses MD simulations to study the coalescence behavior of W/O emulsions stabilized by anionic asphaltene molecules under an electric field. W/O emulsion systems composed of two water droplets with different numbers of anionic asphaltene molecules (*N*) and electric field strengths (*E*) were examined to understand the driving forces of electro-coalescence. The analysis of the behavior of anionic asphaltenes is complex because they can reduce the interfacial tension of water droplets, stabilizing the emulsions, and in turn, they can induce electrostatic effects, increasing the coalescence of the droplets and destabilizing the emulsions. The results obtained in this work may provide important insight into the forces driving the stability of W/O emulsions during oil dehydration by electro-coalescence, where water droplets in emulsions can be stabilized by the presence of anionic asphaltenes obtained at high or moderate water pH.^24^

## 2 Theoretical Background

The exact mechanism by which electro-coalescence occurs is not yet clearly understood because of the complexity of electrostatic and hydrodynamic interactions among swarms of droplets dispersed in a fluid.^11,44^ The mechanism itself is a complex process of coupling many factors involving surface forces, droplet movement, oil–water interface deformation, and the drainage rate of the liquid film between water droplets. The electro-coalescence rate has been related to liquid film thickness, liquid interlayer properties, and multiple interface properties, jointly controlled by inertial force, viscous stress, and surface tension and viscoelasticity.^45–47^ However, a three-step physical phenomenology has been widely accepted when an electric field is applied to the coalescence between droplets in an immiscible liquid medium: (1) close droplets approaching each other, (2) film thinning and/or drainage, and (3) film rupture leading to droplet coalescence.^1,4,48^ During the first step, because of electrostatic polarization (Figure 1a) of the droplets under an electric field, they gradually deform from a sphere to an ellipsoid under the influence of opposite Maxwell stresses.^49–51^ In a relatively weak applied electric field, the droplets deform moderately. For example, the droplets adopt a spheroidal shape, which may be prolate or oblate depending on the transport and electrical properties of droplets and surrounding fluids.^52^ The shape of droplets under the electric field achieves a steady state when the electric stress is balanced by surface tension and viscous stress.^52,53^ Here, the dipole forces are established by the alignment of the polarized water molecules within the droplets (Figure 1a).^54,55^ The polarization of water molecules can bring the droplets closer to each other, separating them by a thin liquid film between their faces. The second step involves thinning this film by liquid drainage to reduce the interfacial area of the system, where the separation pressure between droplets is mainly controlled by two components: van der Waals dispersion force and double-layer force.^56–58^ When the film reaches a certain critical thickness (*h*_c_ (Figure 1b)), each droplet enhances the deformation of the other by short-range electrical forces.^59^ During this second step, any significant disturbance or electrodynamic instability^60,61^ will cause contact between droplets by short-range electrical and van der Waals forces.^59,62,63^ The fluid film eventually breaks due to these two competing forces. Immediately after the contact of the coalescing droplets, a water neck between water droplets is created, serving as a liquid bridge to move charges from one droplet to the other (Figure 1c).^64^

**Figure 1:**
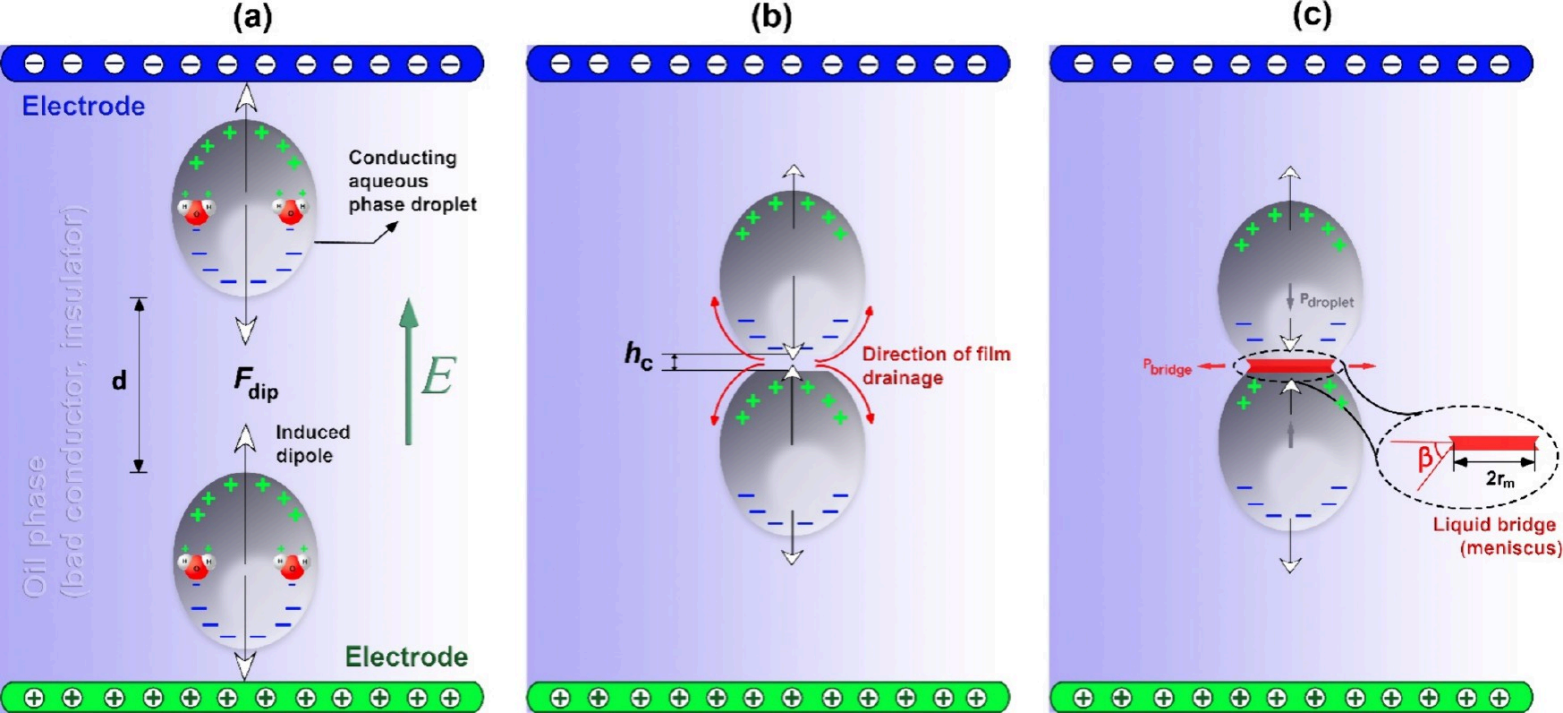
Probable steps of electro-coalescence of a pair of conducting aqueous droplets in a poorly conducting oil phase due to an imposed static electric field *E*. (a) The first step of the coalescence mechanism is induced dipole formation on the droplet surfaces and dipole–dipole interaction (*F*_dip_) between polarized droplets, which creates an interdroplet attractive force and allows their approach. (b) The second step involves the film drainage of the continuous phase (oil) to reach a critical thickness (*h*_c_). Here, a flattened surface between droplets at *h*_c_ is illustrated, in which hydrodynamics and thermodynamic factors act only when the electric field is absent.^62,65^ However, the droplets can deform into sufficiently steep cones or Taylor cones,^60,66,67^ when an electric field is applied. (c) The third step is the formation of a liquid bridge between droplets whose neck curvature is characterized by an angle β and a meniscus radius (*r*_m_) of the interaction of the Taylor cones. The characteristics of the neck curvature of the liquid bridge are related to pressure effects (*p*_droplet_ and *p*_bridge_ (eq 3)).

Liquid bridge formation and coalescence between merging droplets are characterized by a cone angle (β) formed by the intersection of two Taylor cones.^60,68^ β depends on several factors, such as the radius (*a*), surface tension (γ), permittivity (*ϵϵ*_0_), and conductivity (σ) of droplets and the strength of the electric field (*E*) applied to the emulsions, which in turn are related to the electrocapillary number (*ε*_c_ (eq 1)).^60,66^

1 $$\varepsilon_{c}=\epsilon \epsilon_{0}E^{2}a/\gamma$$

2 $$\tan\beta \approx 0.7{\varepsilon_{c}}^{1/2}$$

From a physical point of view, β (eq 2) depends on the balance of electric and capillary forces of colliding droplets and determines the curvature of the liquid bridge. The large curvature (low β) with a small meniscus radius (*r*_m_) (Figure 1c) of the liquid bridge results in strong Laplace pressure driving the droplet fluid into the bridge region,^69^ leading to the expansion of the bridge when Δ*p* > 0 (eq 3).^70^

3 $$\Delta p\approx \frac{\gamma }{r_{m}}\left(\cot\beta -1\right)$$

where Δ*p* = *p*_droplet_ – *p*_bridge_ is the pressure difference between the bulk of the droplet and the meniscus bridge. Wang et al.^71^ showed that *r*_m_ increases with droplet liquid conductivity (σ). Also, numerical simulations show that *r*_m_ has a positive linear relationship with β.^72^ The ratio of *ε*_c_ to the Ohnesorge number ( $\mathrm{Oh}=\mu /\sqrt{\rho \gamma \left(2a\right)}$ , where μ is the bulk viscosity and ρ is the droplet density) has been used to describe the droplet–interface coalescence transition from the viewpoint of flow field evolution and bridge dynamics in coalescence.^73^ With an increase the water droplet conductivity and permittivity, the values of *ε*_c_/Oh can increase, resulting in a partial coalescence–noncoalescence transition.

The complex interplay among the droplets’ charge density, interfacial tension, and local curvature of the liquid bridge governs the coalescence dynamics of moderately charged neighboring droplets.^16^ For successful coalescence, a value of β not exceeding a critical value of 30.8° has been reported for deionized water droplets;^60,68^ however, it could be greater than this critical value for the case of water droplets containing ions.^74,75^ The value of β can dramatically increase when water droplets are about to touch each other due to the electrostatic attraction between cations in one droplet and anions in the counterpart droplet. In this context, the electrostatic force drives ions to migrate through the connecting bridge, resulting in discharge^76,77^ and a partial breakup phenomenon of coalescing droplets.^74^ Moreover, water droplets containing ions can also form sufficiently pronounced cones with β > 45°, in which pinch-off between droplets is observed without coalescence due to the greater pressure in the meniscus bridge than in the bulk of the droplet (Δ*p* < 0 (eq 3)).^68^ The radial (*F*_r_) and tangential (*F*_θ_) components of the electrostatic attraction force between two merging droplets have inferred that coalescence might occur for wider β angles (β < 54.71° and β > 125.19°).^49,78,79^ However, recent experimental studies demonstrated that two conductive droplets suspended in an insulating oil and subjected to an electric field should have a critical cone angle of 23° for noncoalescence.

## 3 Methodology

### 3.1 Setup of Water-in-Oil Emulsion Models

Packmol 18.169 ^80^ was used to construct all W/O emulsion models. Using this package, spherical water droplets of 6 nm diameter containing 3770 water molecules were first constructed. The droplets showed a density equal to 0.997 g cm^–3^, which is the actual density of water under ambient conditions. After the creation of the water droplets, anionic asphaltene molecules (ASP1_8CUV^81,82^ (Figure 2)) were randomly placed within them. The placement of asphaltene molecules inside water droplets can seem unrealistic, since the asphaltenes are a natural part of the crude oil. However, due to their polar groups, asphaltenes can interact through hydrogen bonds with water molecules, showing self-nanoaggregation in solution.^83,84^ In fact, asphaltene molecules can form aggregates, in which water droplets can be trapped within these aggregates. From a simulation perspective, starting from asphaltene configurations in aqueous media, we examined whether such a hydrogen bonding interaction is strong enough to leave asphaltene molecules buried in nanoaggregated states within water droplets when using an electric field. Due to the presence of electric charges and a large fraction of carbon in asphaltene, which is incompatible with water, it should migrate rapidly toward the oil–water interface from bulk water. Thus, the final steady state in our simulations in which the asphaltenes are located on water droplet surfaces before droplet–droplet coalescence is achieved, as demonstrated by our simulations. In this work, the results focus on the dynamics of the liquid bridge between the water droplets when asphaltene molecules are located on their surface. In fact, the asphaltene density profiles on water droplet surfaces estimated just before the initial liquid bridge formation with asphaltenes positioned at the water–oil interface and inside the water droplet are not very different (Figure S1). Moreover, our simulations with asphaltene molecules initially inside water droplets have the advantage of leading to faster droplet–droplet coalescence dynamics compared to those using asphaltenes on the water droplet surfaces, possibly due to steric effects or electrostatic repulsions between asphaltenes on the droplet surfaces at the initial simulation times (Table S1).

**Figure 2:**
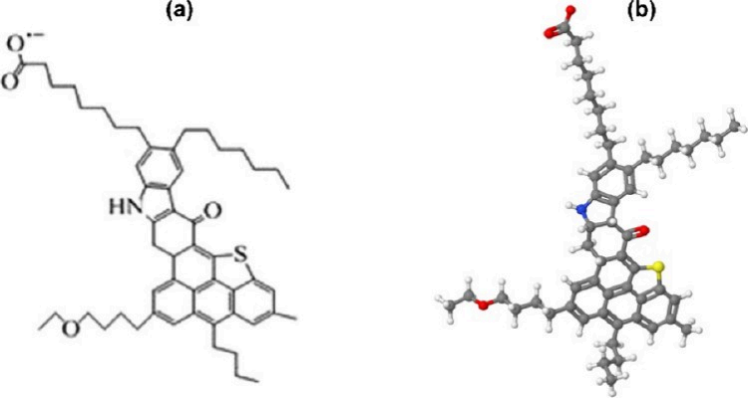
Anionic continental (peri-condensed) molecular model^26,86^ of asphaltene molecule ASP1_8CUV (C_50_H_66_NO_4_S)^25,81,82^ used in our simulations: (a) planar and (b) three-dimensional structures.

Because of the chemical complexity and diversity of asphaltenes, ASP1_8CUV was chosen because this “average molecule”^85^ allows for the successful interpretation of petroleum analytical data.^82^

ASP1_8CUV exhibits some of the commonly postulated structural features of asphaltenes as an average length of five to six carbons for alkyl side chains,^26^ two or more aromatic cores, a H/C ratio of ∼1.1, and a continental-type model.^87^ This asphaltene model is somewhat similar in structure to C5Pe (C_43_H_46_N_2_O_6_), which has been widely used to examine the adsorption of asphaltenes on a silica surface in oil reservoirs^88,89^ and aggregation states in oil–water droplets.^90^ However, CP5e, due to its fused polyaromatic core (perylene type), self-associates more in the *n*-heptane phase than real asphaltenes.^91^ Due to the higher polarity and the presence of sulfur atoms in the model compatible with asphaltene structures, this model was chosen.^25,92^ The number of ASP1_8CUV asphaltene molecules per water droplet (*N*) used in our simulations was 3 and 20, which is within the composition space of asphaltene (about six monomers in aggregates) found in heavy oil^93^ and in agreement with the Yen–Mullins model.^94^ The maximum number of 20 asphaltene molecules per water droplet is sufficient to determine whether asphaltene aggregation occurs on water droplet surfaces. Using combined neutron and X-ray scattering studies of asphaltenes in toluene, flat disk nanoaggregates, which are primary units that can assemble during hierarchical aggregation, were found to contain about this number of asphaltenes.^94,95^

In addition to the W/O models containing droplets with different asphaltene concentrations, water droplets without any asphaltene molecules, “clean droplets”, were created as the control sample. Then, two water droplets with the same composition of asphaltenes were placed in a three-dimensional simulation box (30 nm × 14 nm × 14 nm). The center coordinates of the droplets were (9, 7, 7) and (21, 7, 7) nm (Figure 3). The droplet size was as large as possible to decrease the error caused by the molecular fluctuation. The box size was as small as possible to reduce the computational cost. The composition of the oil, continuous phase, is very complicated.^96^ Therefore, to simplify the simulation, the oil was represented with 26 905 molecules of *n*-hexane (C_6_H_14_), a low-molecular weight hydrocarbon that allows coalescence to occur in a reasonable amount of computational time.^97,98^ Although C_6_H_14_ is used as a simplifying medium to represent crude oil in our simulations, many studies demonstrate that a gas phase, having properties quite different from those of oil, such as nitrogen gas, can satisfactorily reproduce the coalescence behavior of water droplets in an oil-phase environment.^76,97,99,100^ However, although nitrogen gas could be chosen in our systems to accelerate the simulations, we preferred to select an alkane phase, such as *n*-hexane, which has been used in the literature to investigate droplet–droplet electrocoalescence.^97,98^ The insolubility of asphaltenes in *n*-hexane, a molecule with properties similar to those of *n*-heptane, should allow us to visualize, from our simulations, a large number of aggregating monomers on the surface of water droplets, dominated by the π–π interaction, as observed in the molecular simulation of asphaltenes in heptane.^33,101^

**Figure 3:**
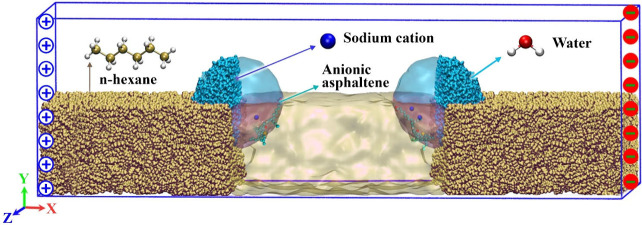
Two water droplets dispersed in oil (*n*-hexane) under a DC electric field (*E*) applied along the *x*-direction. The anionic asphaltene molecules (C_50_H_66_NO_4_S (Figure 2)) within water droplets were drawn in van der Waals notation with VMD.^102^ Oxygen, hydrogen, nitrogen, carbon, and sulfur atoms are represented by red, white, dark blue, light blue (in asphaltenes), beige (in hexane), and yellow spheres, respectively. The asphaltene molecules inside the water droplets inserted by Packmol were practically close to the droplet surfaces (e.g., ∼1 nm for the system with *N* = 3).

The temperatures and pressures of all simulations were 300 K and 1.01 bar, respectively. As in the case of water droplets, the number of C_6_H_14_ molecules was estimated to provide a “real” density under the reference temperature and pressure, thus enabling a stable volume of the box with a saving of computational costs in *NPT* equilibria of emulsion systems.

### 3.2 Simulation and Force Field

All MD simulations were performed with GROMACS 2023,^103,104^ and the GROMOS 54A7 force field was chosen.^105^ The set of parameters of anionic asphaltene (Figure 2) was generated by Automated Topology Builder (ATB),^106,107^ and water molecules were selected as the extended simple point charge (SPC/E) water model.^97,98,108,109^ The SPC/E model adequately captures the properties of liquid water in MD simulations,^110^ thus improving the accuracy of the motion behavior of simulated droplets.^97^ For C_6_H_14_ (*n*-hexane), the united-atom model was adopted, implying that every CH_3_ or CH_2_ group is described as a single interaction site centered at each carbon atom. The interaction between the sites separated by more than three bonds in the same *n*-hexane molecule is described by the Lennard-Jones (LJ) potential.^111^ Overall, the united-atom GROMOS force field performs systematically better than other force fields in reproducing the liquid-phase properties of alkane molecules.^112^

To maintain the electrical neutrality of W/O emulsions, Na^+^ ions were added to the water droplets containing anionic asphaltene molecules. These positively charged ions and negatively charged asphaltene ions can rearrange their positions in the droplet and tend to move and accumulate at the right (Na^+^ ions) or the left (anionic asphaltene ions) edge of droplets, as observed in conducting water droplets with NaCl.^74^ Note that, in the current work, the positive electrode is located on the left side while the negative electrode is located on the right side (Figure 3). At first glance, ignoring the steric hindrance caused by asphaltene molecules adsorbed on the surface of water droplets, the attractive interaction between ions at both edges of water droplets could cause an enhancement in droplet–droplet coalescence. However, in another scenario, the presence of Na^+^ ions may result in few daughter droplets, which are ejected from the coalescing droplet, resulting in partial coalescence,^17,74^ and, perhaps, better adsorption of asphaltenes at the interface, making coalescence difficult.^113^ Here, the Na^+^ ions should act in the water–oil interface by changing the physical properties of the buildup of the interfacial film between the droplets and the continuous phase.^113^ The maximum number of Na^+^ ions (20) added to each water droplet corresponds to ∼0.3 M, which is relatively close to the cation’s molarity (∼0.4 M) used in molecular dynamics studies^17,74^ and experimentally (∼4685 ppm) to investigate the effect of brine chemistry on emulsion stability.^114^ On the other hand, simulations of three Na^+^ ions in water droplets (corresponding to 0.04 M) were used to investigate the effects of the low salinity on droplet–droplet coalescence.^113^ Around this cation’s molarity (∼0.05 M), we examined how the salt dissolved in the aqueous phase induces stability of water/crude oil emulsions due to the aggregation of neutral asphaltenes.

The potential energy of emulsion systems is the sum of the bonded energy and the nonbonded energy, in which most MD calculation occurs in the computation of the nonbonded energy.^31^ The nonbonded interaction energy includes electrostatic interaction (*E*_AB_^elec^) and van der Waals (vdW) interaction (*E*_AB_^vdw^), as shown in eq 4.

4 $$E_{AB}^{\mathrm{non‐bonded}}\left(r_{\mathrm{AB}}\right)=E_{\mathrm{AB}}^{\mathrm{elec}}\left(r_{AB}\right)+E_{\mathrm{AB}}^{\mathrm{vdw}}\left(r_{AB}\right)=\frac{q_{A}q_{B}}{4\pi \epsilon_{0}r_{AB}}+\frac{C_{12}^{AB}}{{r_{AB}}^{12}}+\frac{C_{6}^{AB}}{{r_{AB}}^{6}}$$

where *q*_A_ and *q*_B_ are atomic charges, *ε*_0_ is vacuum dielectric constant, *r*_AB_ is the distance between atoms A and B, and *C*_12_^AB^ and *C*_6_^AB^ are the Lennard-Jones (LJ) parameters between atoms. The interaction LJ parameters in GROMOS force fields^115^ are determined by transformation rules of cross-term LJ parameters (eqs 5 and 6).

5 $$C_{12}^{AB}=\sqrt{C_{12}^{AA}C_{12}^{BB}}$$

6 $$C_{6}^{AB}=\sqrt{C_{6}^{AA}C_{6}^{BB}}$$

where *C*_12_^AA^, *C*_12_^BB^, *C*_6_^AA^, and *C*_6_^BB^ are the LJ parameters of the same atom.

Periodic boundary conditions (PBC) were used to make the boundary displacement continuous in all directions of the simulation box.^116^ The choice of PBC is important to approximate a large (infinite) system using a small part called the unit (replicas) cell. This approximation can cause artificial interactions between periodic replicas, which can lead to overestimation of long-range interactions, especially in charged systems with a nonuniform electric field distribution, as water droplets laden with anionic asphaltenes, thus altering conformations and transport properties.^117^ Due to this limitation, we analyze the simulation results (e.g., diffusion of species and conductivity (sections 4.2.5 and 4.2.6) and compare them to the actual system to ensure that the results are accurate and reliable.

The first step in MD simulations was energy minimization to eliminate atom clashes. The energy minimization for each system is conducted by the steepest descent algorithm^118^ until the maximum force on any atom is less than 100 kJ mol^–1^ nm^–1^. Before the external electric field was applied, two equilibration stages were carried out on the W/O emulsions to reach the preset temperature and pressure values. The first stage consisted of equilibration under an *NVT* (constant number of molecules, volume, and temperature) ensemble for 100 ps. The velocity-rescaling (V-rescale) method^119^ was used for the thermostat with a damping constant of 0.1 ps. The system’s temperature in this equilibration must be 300 K. The second equilibration stage was carried out under an *NPT* (constant number of molecules, pressure, and temperature) ensemble for 1000 ps to bring the emulsion to the desired pressure (1.01 bar). Here, the barostat based on the stochastic cell rescaling (C-rescale) method^120^ was employed to control the pressure of the system. The time constant used for the barostat was 2.0 ps. In both equilibration stages, the time step was 2 fs, and the trajectories were collected at 1.0 ps intervals for further analysis. The SETTLE algorithm^121^ was used to constrain the bond lengths and angles of water molecules, whereas the bonds of *n*-hexane molecules were constrained using the LINCS algorithm^122^ to reduce the number of degrees of freedom and accelerate the simulation. From these two equilibration stages, the emulsion models are expected to show the actual densities of the different phases under room conditions.

After the equilibrium systems were prepared, the temperature of the surrounding oil was maintained at 300 K by rescaling the velocities of the *n*-hexane molecules. Then a homogeneous electric field along the *x*-direction^123^ was applied to the system for studying electro-coalescence (Figure 3). The electric field strength (*E*) was 0.3–0.6 V/nm, which is an acceptable range for the coalescence and noncoalescence to occur in conducting and pure water droplets.^76,124^ A critical value of *E* (∼0.5 V/nm) for the studied systems, regardless of factors such as conductivity, type, and diameter of water droplets, is expected to be in this range, which has been used to study the coalescence of charged water droplets.^52,75,98^ The *E* values are low enough for the asphaltenes to be adsorbed at the interface without being removed toward the *n*-hexane phase.^32^ Thus, an additional electrical force (*F*_i_) is imposed for each atom with a *q*_i_ charge in the W/O emulsions given by *F*_i_ = *q*_i_ *E*. The Parrinello–Rahman (PR) method^125^ was employed to adjust the pressure of this production stage with a coupling constant of 5.0 ps. The simulation time was 5000 ps with a time step of 1 fs to obtain accurate velocity and coordinates in the temperature- and pressure-controlled systems. At each *E*, the simulations were replicated three times to achieve statistical validity and to produce robust findings.

In all simulations, the Newtonian equation of motion was integrated by the leapfrog algorithm.^126^ Long-range electrostatic interactions were handled using the particle mesh Ewald (PME) algorithm,^127^ with a cutoff distance of 1.0 nm, whereas the short-range van der Waals interaction was described by the Lennard-Jones potential, with a cutoff distance of 1.0 nm.^128^ The graphics of motion trajectory were obtained using VMD (version 1.9.2).^102^

### 3.3 Model Validation

The current simulations are compared to the results of a W/O system studied by Guo and He,^124^ as shown in Figure 4. The comparison shows good agreement for dynamic droplet behaviors between simulations and experiments, e.g., a complete coalescence at a low electric field strength (*E*). It should be noted that, in the present simulations, the order of magnitude of the applied electric field strength and the time scale required for coalescence deviate from the experiments. Note that *E* in our simulations is about 3 orders of magnitude higher than in experiments (Figure 4), because the effect of a low *E* will be overshadowed by the molecules’ thermal motion in MD simulations.^17,76,129,130^ On the other hand, the present simulations also adopt nanoscale droplets because of the limitation of MD computations. Taking into account these limitations, the present MD simulations reproduce the phenomena observed in the macroscopic experiments, and hence, they do not hamper our understanding of electro-coalescence at the molecular level. MD simulation is an accurate method,^131^ which can give useful information on the nanoscale’s physical process of droplet coalescence.

**Figure 4:**
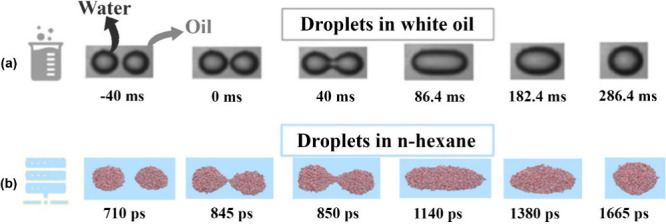
Coalescence process of two water droplets without asphaltenes: (a) experiment (white oil; *E* = 0.167 × 10^3^ kV/m; *d* (droplet diameter) = 1.72 mm)^124^ and (b) simulation (*n*-hexane; *E* = 0.5 × 10^6^ kV/m; *d* = 2*a* = 6 nm).

### 3.4 Trajectory Analysis

#### 3.4.1 Solvent Accessible Surface Area

To determine when the droplets have completely coalesced, the solvent accessible surface area (SASA)^132^ of water droplets was monitored during simulations. Some studies^31,97^ showed that the SASA calculation is a reasonable technique to estimate coalescence time where there is a deformation of droplets under the polarization of an external electric field. In other words, when the water molecules are subjected to the force of the electric field, they migrate and a tensile deformation of the droplet first occurs. Consequently, the SASA values of the two droplets increase. Then, when the two droplets come into contact, they begin to merge, showing a decrease in the total SASA with respect to the sum of the surface area of each water droplet. Once the droplets have completely merged, SASA will remain stable.

#### 3.4.2 Potential Energy and Hydrogen Bonds of Water Droplets

Other criteria for determining when coalescence occurs are the profiles of the potential energy and hydrogen bond (H-bond) number of the water droplets over the simulation time. Such criteria are based on the fact that when two water droplets approach each other during coalescence, there is an increase in the number of hydrogen bonds (NHB), with a decrease in potential energy, which is mainly caused by a decrease in electrostatic energy.^76,133^ After coalescence is complete, the mixing rate of the water molecules in the two droplets is reduced, and the increasing trend in the number of hydrogen bonds gradually slows, thus affording a maximum in the NHB profiles with simulation time. Li et al.^31^ showed that the NHB between droplets can also be used to determine the droplet coalescence time efficiently.

#### 3.4.3 Radial Distribution Functions

To provide a deeper understanding of the interaction among different molecules in the emulsions, radial distribution functions (RDFs or *g*(*r*))^134^ for equilibrium structures achieved during *NPT* simulation were analyzed under the application of a force field. From the motion trajectory of atoms, *g*(*r*) (eq 7) can be computed with VMD^102^ using the radial pair distribution function module.

7 $$g\left(r\right)=\frac{n_{\mathrm{avg}}\left(r\right)}{\left(\frac{4\pi }{3}\right)\left({\left(r+\Delta r\right)}^{3}-r^{3}\right)\rho_{\mathrm{avg}}}$$

where *n*_avg_(*r*) is the average number of atoms around an atomic center between *r* and Δ*r*. $n_{\mathrm{avg}}\left(r\right)/\left[\left(\frac{4\pi }{3}\right)\left({\left(r+\Delta r\right)}^{3}-r^{3}\right)\right]$ is the local density of atoms in a shell of thickness Δ*r*, and *ρ*_avg_ is the average bulk density. Thus, *g*(*r*) is a function representing the probability of finding an atom in a shell Δ*r* within a distance *r* of another atom chosen as a reference point. In this study, *g*(*r*) was used to calculate interactions between the interfacial water molecules and asphaltene molecules. The results of *g*(*r*) calculations allow us to conclude that fundamental intermolecular interactions are π–π stacking, hydrogen bonds, and other noncovalent bonds between asphaltenes and water^28,41,134^ as a function of electric field strength (*E*) applied to the emulsion systems.

#### 3.4.4 Dipole Moments, Charge Density Distribution, and Electrostatic Forces

When the distance between the inner faces of droplets is small, the interstitial electric field is much stronger than the field anywhere in the bulk phase. Both charged interfaces induce mirror charges on each other in the droplets.^16^ A strong electric field causes dielectrophoretic attraction and shape deformation, because of the strong local electric stresses. Therefore, despite the lower interfacial tension provided by asphaltenes on a water droplet surface, which affords a larger thin film with stronger resistance to coalescence,^135^ a larger deformation and higher curvature to the inner faces of the droplet pair can occur when an electric field is applied. From an electrostatic point of view, in these studied systems, the convection of charges to the high-curvature part from the rest of the droplet interface can be important, as it ramps up the local charge density, enhancing droplet–droplet coalescence. In this complex interaction of hydrodynamic and electrical factors, the estimation of the charge density distribution curve and the electric charges between the inner faces of the droplets derived from the curves are required, which can clarify the mechanism of dipole–dipole coalescence under mesoscopic and macroscopic conditions.^4^

The electric charge (*z*, equivalent to the number of protons) created on the surface of the droplets and the electrostatic force between them can be derived from the integration of the charge density distribution (ρ­(*x*) (Figure 5)) along the droplet in the *x*-axis direction, as follows:

8 $$z=4\pi \int_{x_{0}}^{x_{1}}\rho \left(x\right)x^{2}\;dx$$

where the integration limits span a slice thickness (1.5 nm)^136^ of the droplet corresponding to the surface layer of excess charge. For instance, for droplet 1, *x*_1_ is located at its leading edge (Figure 5), whereas *x*_0_ = *x*_1_ – 1.5 nm. On the other hand, in the case of dipole–dipole interaction between two similar spherical droplets aligned with the applied electric field, the following equation can be used to estimate the electrostatic force of dipoles at a given value of *S*:^55^

9 $$F_{\mathrm{dip}}=\frac{-12\pi \omega^{2}\epsilon_{m}E^{2}{r_{1}}^{3}{r_{2}}^{3}}{S^{4}}\left(3K-1\right)$$

where *S* is the separation distance between the center of the droplets and *ϵ*_m_ is the medium permittivity (1.664 × 10^–11^ C^2^ N^–1^ m^–2^). *S* was estimated by tracking the distance between the mass center of droplets in the atomic trajectory motions just before (70 ps) the formation of the liquid bridge between merging droplets. Taking into account this simulation time, the found value of *d* (0.6–5.6 nm (Figures S5–S7)) is somewhat larger than that of *h*_c_ (0.14 nm (Note S2)) calculated for our W/O systems. At these distances (*d*) relatively close to *h*_c_, film thinning/drainage occurs (second step of coalescence), and the coalescence of droplets shows a strong dependence on short-range electrical forces, as expressed by eq 9. The Clausius–Mossotti factor (ω) in eq 9 is defined as follows:^55^

10 $$\omega =\frac{\epsilon_{d}-\epsilon_{c}}{\epsilon_{d}+2\epsilon_{c}}$$

and *ϵ*_d_ (=78.4)^138^ and ϵ_c_ (=1.88)^139^ are dielectric constants of water and oil (*n*-hexane in our study), respectively. Coefficient *K* can be expressed as shown in eq 11.

11 $$K=1+\frac{\omega {r_{1}}^{3}S^{5}}{{\left(S^{2}-{r_{2}}^{2}\right)}^{4}}+\frac{\omega {r_{2}}^{3}S^{5}}{{\left(S^{2}-{r_{1}}^{2}\right)}^{4}}+\frac{3\omega^{2}r^{1}{r_{2}}^{3}\left(3S^{2}-{r_{1}}^{2}-{r_{2}}^{2}\right)}{{\left(S^{2}-{r_{1}}^{2}-{r_{2}}^{2}\right)}^{4}}$$

**Figure 5:**
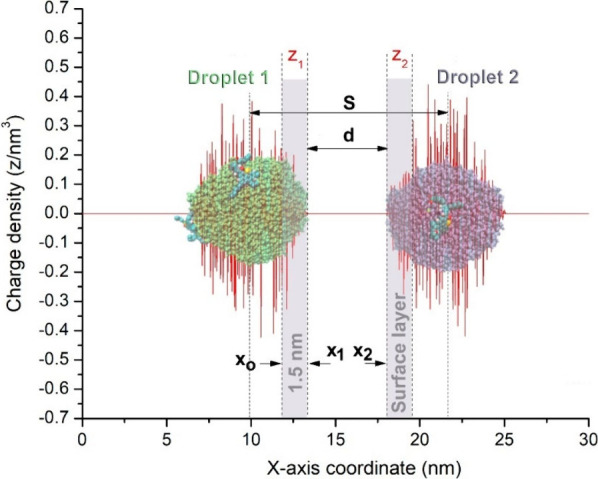
Distribution of charge density (ρ­(*x*) in red lines) of two coalescing droplets under a DC electric field (*E*) applied along the *x*-axis. Here *z*_1_ (for water droplet 1) and *z*_2_ (for water droplet 2) (eq 8) were the estimated electrical charges for a surface layer of excess charge in each droplet with a thickness equal to 1.5 nm.^136^ Such a surface layer thickness is reasonable if one takes into account the fact that the hydrodynamic radius of small asphaltene molecules, like those considered here, is about 1 nm.^137^ *S* accounts for the distance between droplet centers just before creating the liquid bridge (Figure 1b) and is approximately 1 order of magnitude larger than *h*_c_.

Considering that ω = 0.93 (eq 10), the radii of the droplets (*r*_1_ and *r*_2_) are equal (*r*_1_ ≅ *r*_2_ = *a* = 3 nm), and the *S* values provided in Table S4, *K* values were estimated (Table S3). From these values, *F*_dip_ was then calculated using eq 9. *F*_dip_ could be considered as one of the short-range forces affecting the fate of two polarized droplets within the dielectric medium.^64^ The increase in *F*_dip_ on the surface of the water droplets leads to the creation of an internal electric field that counteracts the external one, thus reducing the electric field within the droplets.^140^

#### 3.4.5 Ionic Concentration at the Interface between Water Droplets

Since the electrocapillary number (*ε*_c_) and β are influenced by two different phenomena (conductivity (σ) and interfacial tension (γ))^66,141^ and, in turn, these are given by the number of asphaltenes (*N*_asph_^inter^) that accumulated at the interface between the inner faces of the water droplets, it is important to estimate *N*_asph_^inter^. This variable was estimated from the mass density distribution along the *x*-axis coordinate (ρ­(*x*)) using the function *gmx density* ^142^ in GROMACS. Also, ρ­(*x*) was estimated for sodium cations within water droplets, which can reveal possible interactions between them and anionic asphaltenes on water surfaces. The relative amount to the total for each ionic species, anionic asphaltenes and sodium cations, was calculated by integrating ρ­(*x*) using the same integration limits (*x*_0_, *x*_1_) as in the estimation of electrical charges (Figure 5). Because few asphaltenes can accumulate at the interface between leading edges of water droplets, especially with the system at the lowest *N* (=3), we extend the integration range taking into account the deformation ratio (*D* (Table S2)) of each water droplet at the integration limits. For example, when calculating *N*_asph_^inter^ for a W/O system with *N* = 3 for droplet 1 (*N*_asph_^inter^)^1^ and droplet 2 (*N*_asph_^inter^)^2^, the following equations are used:

12 $${\left(N_{\mathrm{asph}}^{\mathrm{inter}}\right)}^{1}=3\frac{\int_{x_{1}-1.5-6D_{1}}^{x_{1}}\rho \left(x\right)x^{2}\;dx}{\int_{-\infty }^{+\infty }\rho \left(x\right)x^{2}\;dx};{\left(N_{\mathrm{asph}}^{\mathrm{inter}}\right)}^{2}=3\frac{\int_{x_{2}}^{x_{2}+1.5+6D_{1}}\rho \left(x\right)x^{2}\;dx}{\int_{-\infty }^{+\infty }\rho \left(x\right)x^{2}\;dx}$$

where *x*_1_ and *x*_2_ are the distances (denoted by vertical dashed lines in Figure 5) at which are located the leading edges of water droplets 1 and 2, respectively. This distance was used to estimate electrical charges (section 3.4.4) and precisely corresponds to 70 ps of simulation time, just before forming the liquid bridge in the colliding droplets. At this simulation time, the deformation ratio of droplets (*D*_1_ and *D*_2_) was evaluated under an electric field (Table S2) and then considered in integration limits. The total number of asphaltenes molecules that accumulated between leading edges of colliding water droplets (*N*_asph_^inter^) is therefore the sum of (*N*_asph_^inter^)^1^ and (*N*_asph_^inter^)^2^.

#### 3.4.6 Ionic Diffusion and Conductivity

To calculate the self-diffusion coefficient of species involved in coalescence (asphaltenes (*D*_asp_) on the surface of droplets, sodium cations (*D*_Na_), and water molecules (*D*_water_)), the structural configuration at 70 ps of the *NPT* ensemble was extracted. This configuration was used to start a 1 ns production run in the *NVT* ensemble, which is a suitable thermodynamic ensemble to calculate transport properties.^143^ *D* (eq 13) can be estimated from the slope of MSD versus lag time τ.^144^

13 $$D_{i}=\underset{t\to \infty }{\lim}\frac{1}{6t}\left\langle {\left|r_{i}\left(t\right)-r_{i}\left(0\right)\right|}^{2}\right\rangle =\frac{1}{6t}MSD_{i}$$

where *r*_*i*_ and MSD_*i*_ are the position and mean square displacement of species *i*, respectively. *D*_*i*_ is determined by dividing the slope of the curve of MSD_*i*_ versus simulation time by 6 (Figure 6).

**Figure 6:**
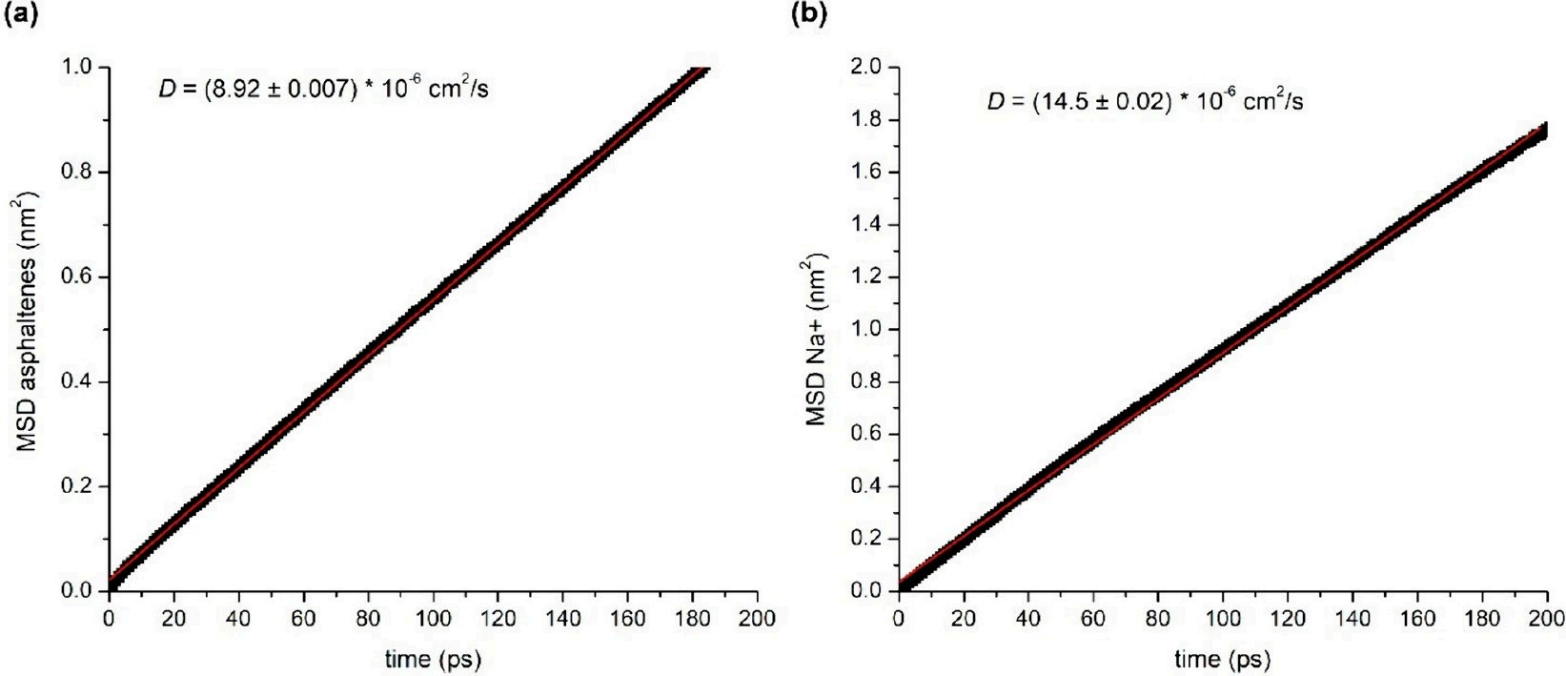
Curves of the mean square displacement (MSD) for (a) asphaltenes and (b) sodium cations vs simulation time determined for W/O emulsions with *N* = 3 at 0.3 V/nm. The slope of the straight line allows us to calculate the self-diffusion coefficient (*D*) of the species with eq 13.

Knowing the self-diffusion coefficient (*D*_*i*_) for each conductive species *i* participating in the coalescence, its electrical conductivity (*σ*_*i*_) was computed by the Nernst–Eisntein equation:^145^

14 $$\sigma_{i}=\frac{{q_{i}}^{2}C_{i}}{k_{B}T}D_{i}$$

where *q*_*i*_ and *C*_*i*_ are the charge and concentration (mol/cm^3^) of species *i*, respectively, *k*_B_ is the Boltzmann constant, and *T* is the temperature. The total conductivity of the system (here considered the conductivity of the dispersed phase) is given by anionic asphaltenes and sodium cations in the water droplets, which was estimated by ∑_*i*_ *σ*_*i*_.

### 3.5 Limitations and Assumptions in MD Simulations

The MD simulations in the current work show simplifications of reality and are thus inherently limited in their ability to perfectly replicate experimental findings. The following limitations were found.

#### 3.5.1 Structure of the Chosen Asphaltene Model

In order to successfully simulate systems containing asphaltenes, it is vital to have an accurate description of the molecular structure of asphaltenes. This is a challenging step because asphaltenes constitute an entire class of molecules and not an individual species. There are very few systematic methodologies for proposing model structures of asphaltenes, and the proposal of models is rather based on experimental information that is only marginal and in many cases inconclusive and contradictory.^33^ Furthermore, it is known that asphaltenes have a wide multimodal distribution of sizes. Therefore, it is unlikely that only one average molecule can be a valid descriptor for such a complex system. The polydispersity in the asphaltene molecular structures is of great importance for the prediction of aggregation structures, which is driven by a sum of correlated contributions from aromatic cores, aliphatic chains, or heteroatoms.^146^ However, for practical reasons, any study is bound to be limited to a small number of prototypical molecules, mainly as a consequence of the relatively limited computational power available coupled with the uncertainty and experimental challenge of describing asphaltenes. Here, we chose ASP1_8CUV as the asphaltene model, which is based on quantitative molecular representation (QMR),^82^ to remove some of the empirical questions. The model can detect an aggregation number of asphaltene molecules of ∼3–5 at the interface between the inner faces of the water droplets (Table 3), in agreement with the “Yen–Mullins” model^3^ but in disagreement with the X-ray and neutron scattering results, where the nanoaggregates may be larger.^94,95^ However, the dimers of the asphaltene model used in the current work bind in a face-to-face manner at a distance of 3.9 Å (Figure S9a) similar to that found in other asphaltene models in heptane (3.5 Å).^147^

#### 3.5.2 Solvent (*n*-hexane) Used to Mimic the Complex Mixture of Hydrocarbons and Other Compounds Found in Crude Oil

Although we selected *n*-hexane as a solvent to aid in the aggregation and precipitation of asphaltenes on the surface of water droplets, it is experimentally recognized that crude oil is composed of a wide mixture of hydrocarbons, where the ratio of toluene (good solvent) and aliphatic hydrocarbons (poor solvent or precipitant) can induce asphaltene aggregation/flocculation.^148^ Hexane, being a nonpolar solvent, is frequently used to separate and characterize asphaltenic subfractions due to the strong tendency of asphaltenes to aggregate and its solubility characteristics.^148,149^ This solvent has recently been used in MD studies^97,98^ to unravel the molecular mechanism of droplet electrocoalescence at the oil–water interface under a DC electric field, in the absence of asphaltenes. Simulating large and complex systems such as crude oil can be computationally demanding. In fact, many studies use nitrogen gas, which has properties very different from those of oil, to accelerate simulations of the coalescence of conductive droplets under an electric field.^76,97,99,100^

#### 3.5.3 Placement of Asphaltenes within Water Droplets

Since asphaltenes are a natural part of crude oil, from a simulation point of view we expected to place them into the solvent model (e.g., *n*-hexane) and not within the water droplets. The placement of asphaltenes into water droplets is a simulation artifact intended to demonstrate that asphaltene’s model can migrate toward the water droplet surface, despite having polar groups capable of forming hydrogen bonds, and it does not remain in the aqueous phase during application of an electric field. On the other hand, because the initial charged configurations of asphaltene molecules are very close to the water–oil interface (Figure 3) and, to some extent, the repulsive negative charges of asphaltenes are shielded by surface water molecules in the droplets, the droplet–droplet coalescence onset time is drastically reduced until all asphaltenes have migrated to the interface. In other words, if asphaltene molecules were initially positioned on the water droplet surfaces, the high negative charge on the surface and the steric effect of asphaltenes would prevent tracking the drop-by-drop coalescence on short time scales in MD simulations, especially for W/O emulsions with high *N*. Alternatively, if the asphaltenes were placed into the *n*-hexane phase, due to their insolubility, aggregates or clusters could form, making it difficult for the asphaltenes to diffuse toward the water droplet surface, thus increasing the calculation time for the equilibrations.

#### 3.5.4 Ions in the Aqueous Phase of Water Droplets

Another limitation of this work is the consideration of a single ion within the water droplets. From reality, it is expected that many types of ions (Cl^–^, SO_4_^2–^, Mg^2+^, Na^+^, and Ca^2+^) can be present inside the water droplets. The type and concentration of ions in a water droplet significantly affect emulsion stability by influencing droplet size, interfacial properties, and droplet interactions.^150^ For example, here the effect of ion concentration on water droplet size is not taken into account, and it is assumed that, regardless of the number of Na^+^ cations, the droplets maintain the same size (6 nm in diameter). In this work, the Na^+^ cation was considered as a model ion because the hydration number should be small due to the small atomic radius; therefore, the restriction provided by the surrounding water molecules is weak, and the formation of secondary drops (if it occurs) can be detected during the breaking of liquid bridges.^75^ In general, increasing the number of ions in water droplet simulations tends to increase the computation time because more pairwise interactions need to be calculated.

## 4 Results and Discussion

### 4.1 Minimization and Equilibration of the Water-in-Oil Emulsions

The potential energy minimization of two water droplets containing different amounts of asphaltenes per droplet (*N*) in oil (*n*-hexane) is depicted in Figure 7. Minimization was stopped with a maximum force of around 89.6 kJ mol^–1^ nm^–1^ and a negative potential energy between −1.25 × 10^–6^ and −1.28 × 10^–6^ kJ/mol for all W/O systems. The negative values and magnitudes of the potential energy are comparable with those found for proteins in water, which indicates that the W/O systems are stabilized by attractive interactions.^151^ The slight decrease in potential energy with *N* could indicate some stability achieved by the droplets because of stronger attractive interactions between water and asphaltene molecules by hydrogen bonds, which will be demonstrated below.

**Figure 7:**
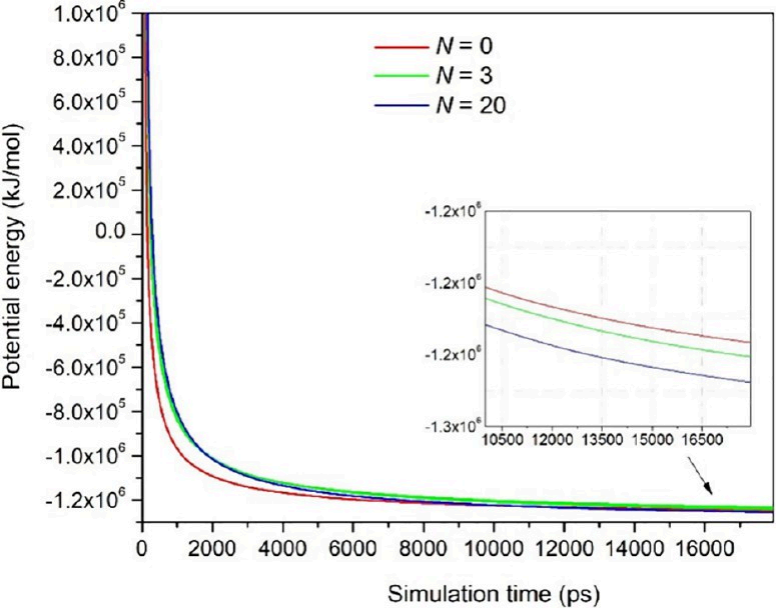
Minimization of the potential energy in W/O emulsions with simulation time. The data represented in this figure show a large subset of the minimization results. The inset shows potential energy data at the final times of the simulation corresponding to W/O emulsions with different numbers of anionic asphaltene molecules per water droplet (*N* = 0, 3, and 20).

After minimization, the emulsion systems were equilibrated to bring the temperature (300 K), pressure (1.01 bar), and density to ambient conditions, as shown in Figure 8. As one can see in Figure 8a, the temperature of all systems reaches an average value of 300 K, which is close to that at room temperature. On the other hand, under *NPT* equilibration the pressure of these systems approaches average values close to 1 bar (Figure 8b), whereas their densities (Figure 8c) achieve average values between 0.623 and 0.629 g cm^–3^, which are close to the experimental density found for *n*-hexane (0.66 g cm^–3^).^152^ The similarity of the system density to that of *n*-hexane is reasonable considering that *n*-hexane is the major component in the W/O emulsions studied here. Curiously, we observed that the density of the emulsions increases with *N* (Figure 8c). This fact, as mentioned above, could be related to stronger attractive interactions between water and asphaltene molecules, leading to denser systems.

**Figure 8:**
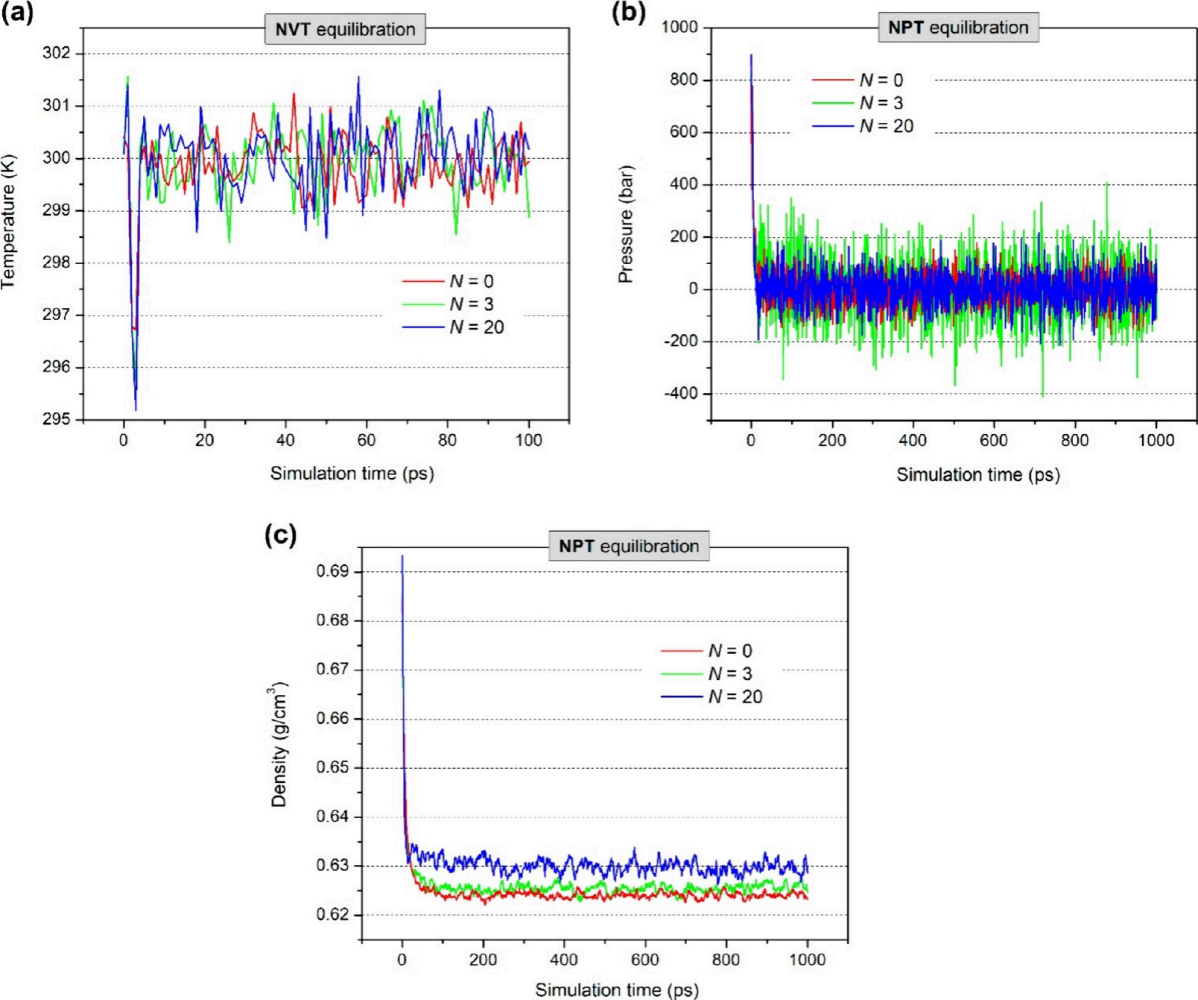
Control variables (temperature, pressure, and density) monitored during (a) *NVT* and (b and c) *NPT* equilibration for W/O emulsions with different numbers of anionic asphaltene molecules per water droplet (*N* = 0, 3, and 20).

All of the above results show that W/O emulsions containing different *N* values are well equilibrated under ambient conditions (300 K and 1.01 bar). Then, different electric field intensities (*E*) can be applied to W/O emulsions to study droplet–droplet coalescence.

### 4.2 Droplet–Droplet Coalescence

#### 4.2.1 Determination of the Electro-coalescence Time by the SASA

The SASA profiles with simulation time for W/O emulsions at different values of *N* (0–20) and *E* (0.3 – 0.6 V/nm) are shown in Figure 9. From profiles of three independent simulation experiments were estimated the values of the coalescence onset time (*t*_c_) for the different studied systems, as shown in Table 1.

**Table 1:** Droplet–Droplet Coalescence Onset Times (*t*_c_, ps)^a^ at Different Electric Field Strengths (*E*) and Numbers of Asphaltenes per Droplet (*N*) in W/O Emulsions

*E* (V/nm)	*N* = 0	*N* = 3	*N* = 20
0.3	ND^b^	ND	ND
0.4	ND	1560 ± 940	ND
0.5	1477 ± 627	902 ± 122	2970 ± 500
0.6	570 ± 185	432 ± 92	627 ± 33

a Values of *t*_c_ were determined by averaging three independent simulations.

b ND means not defined because droplet–droplet coalescence was not detected (Figures 9 and 12).

**Figure 9:**
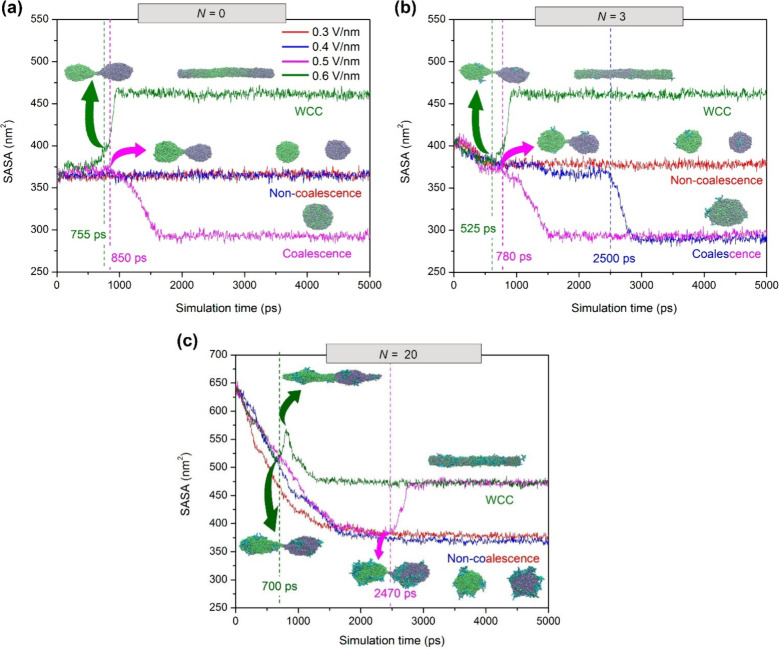
Profiles of the solvent accessible surface area (SASA) of droplets with simulation time for W/O emulsions at different electric field strengths (*E* = 0.3–0.6 V/nm) and numbers of anionic asphaltene molecules per water droplet (*N* = 0, 3, and 20). Insets are simulation snapshots of water droplets with asphaltene molecules corresponding to electro-coalescence behaviors: (1) noncoalescence, (2) complete coalescence, and (3) formation of a water chain configuration (WCC) between electrodes. Water droplets are colored green and lime, whereas asphaltene molecules on droplet surfaces are colored cyan. The vertical dashed lines indicate the onset time (*t*_c_) of droplet–droplet coalescence. The results shown correspond to a replication of the simulation experiments.

Figure 9 reveals three well-defined coalescence behaviors as a function of electric field strength (*E*) and number of asphaltene molecules per water droplet (*N*): (1) noncoalescence, (2) complete coalescence, and (3) formation of a water chain configuration (WCC)^153–155^ between electrodes. The latter behavior impairs the dewatering efficiency of crude oil.^64,156^ In general, the noncoalescence process seems instead to occur at low *E* and high *N*. As also shown in Figure 9, the application of an electric field to W/O emulsions with *E* ≤ 0.3 V/nm does not cause merging between water droplets, mainly due to their insufficient dipole–dipole forces, as discussed below (section 4.2.8). The complete droplet–droplet coalescence occurs because of an interplay of different factors, including the variation of *E* and *N*. At a moderate *E* value (between 0.4 and 0.5 V/nm) is observed a complete coalescence of the water droplets, which is rather favored depending on *N* (Videos S1–S3). It appears that an increase in *N* from 0 to 3 at *E* = 0.5 V/nm somewhat decreases the coalescence onset time (*t*_c_) from 1477 to 902 ps (Table 1).

Moreover, water droplets with *N* = 3 show a complete droplet–droplet coalescence at a lower electric field strength (e.g., *E* = 0.4 V/nm (see the blue curve in Figure 9b)), which is not observed for emulsions with clean water droplets (Figure 9a). The above results reveal that coalescence can be favored when water droplets are laden with anionic asphaltenes at some degree of concentration, and under this condition, the critical *E* (*E*_c_) of the coalescence process is reduced.^16^ The improvement in coalescence with anionic asphaltenes at moderate *E* and low *N* is in consonance with a study reported by Mhatre et al.,^16^ in which W/O emulsions with neutral asphaltene-laden droplets facilitated the electro-coalescence process compared to those with clean water droplets. As also pointed out by Chen et al.,^157^ low ionic concentrations can lead to dipole polarization of droplets, decreasing the coalescence time, which will be discussed below (section 4.2.7).

Analyzing the SASA profiles at *E* = 0.4 V/nm (Figure 9b,c), one can see that a further increase in *N* by 17 molecules of asphaltenes hinders droplet–droplet coalescence. As we will discuss below (section 4.2.4), this is due to the larger amount of asphaltenes accumulated by a self-aggregation effect at the adjacent interfaces of the droplet pair (Video S3).^21,22,24,27,28^ All of the above findings allow us to infer that a moderate number of ionic asphaltene molecules that accumulated on the surfaces of the water droplets (e.g., *N* = 3) favors Coulombic attractions between the droplet pair during coalescence, and such attraction might have a more significant effect than the interfacial stabilization by the self-aggregation of asphaltenes, which opposes the drainage of the film and breakage of the thin film. This effect will be discussed in sections 4.2.7 and 4.2.8 based on the dipole–dipole forces acting on the water droplets.

Unlike the complete coalescence observed at moderate *E* and low *N*, the WCC phenomenon is best noted when *N* and *E* are both increased (olive and magenta curves in Figure 9c). Under this condition, the surface charge per water droplet is quite large, and the electrostatic force between them is attractive in nature (e.g., −460 e^2^ for the system at *E* = 0.6 V/nm and *N* = 20 (Table S4)). Here, despite the electrostatic attraction between water droplets being favored, the self-aggregating effect of ionic asphaltene molecules on their surfaces is increased, making droplet–droplet coalescence difficult (section 4.2.4). The aggregation effect of asphaltenes acts together with the high degree of stretching of water droplets to give rise to the formation of the WCC extended from one electrode to another at the highest values of *E* (e.g., 0.5–0.6 V/nm (Video S4)).^1,153^ Indeed, such a formation can even be achieved at lower *E* values with an increase in *N* (see the SASA profiles at *E* = 0.5 V/nm in panels b and c of Figure 9), because there is an increase in the electrical charges on the water droplet surface (section 4.2.6), leading to a more severe deformation on the leading edge of each droplet.^76^ Beyond WCC formation, the generation of secondary tiny droplets or rebound at high *E* ^74^ was not observed at high *N* despite the high degree of stretching of conductive droplets^158^ and the high concentration of sodium cations dissolved in water droplets.^75,159^ Perhaps the large elasticity of water droplets containing long chain structures of asphaltene molecules (Figure 2) does not allow secondary droplets to form easily.^44^ Another explanation may be based on the insufficient kinetic energy of the dissolved ions (in our case sodium cations), which are unable to overcome the surface energy of the coalescing droplets and, therefore, cause the formation of daughter droplets.^17^

To better understand the forces exerted on the water droplets laden with anionic asphaltene molecules, other factors must be studied, such as hydrogen bonds, the electrostatic potential, the cone angle of the liquid bridge, deformation of water droplets, and ionic conductivity, which in turn are related to the distribution of the charge and motion of ions on water droplet surfaces.

#### 4.2.2 Hydrogen Bonding and Potential Energy

Figure 10 shows the profiles of the number of hydrogen bonds (H-bonds) of the W/O systems studied here. As mentioned above, in the case of complete coalescence between water droplets, an increase in the total number of H-bonds between them is expected when droplet–droplet coalescence begins.^98^ Here, it is anticipated that the values of electrostatic potential are more negative, indicating that there is a strengthening of electrostatic attractions during fusion between droplets, as shown in Figure 11. The above description is physically reasonable based on the fact that during the fusion of water droplets, new attractive Coulomb interactions are created by H-bonds between water atoms of different colliding droplets, as observed between the H_2_O in a droplet and the H_2_O in bulk water.^98^ We observe from our simulations that the major contribution to the change in the system energy is due to Coulomb interactions (Figure S2).^133^ In other words, the electrostatic attraction interactions between water molecules play a more dominant role than the van der Waals interactions during droplet–droplet coalescence, as shown in the electro-coalescence of water droplets in W/O emulsions containing a surfactant.^31^

**Figure 10:**
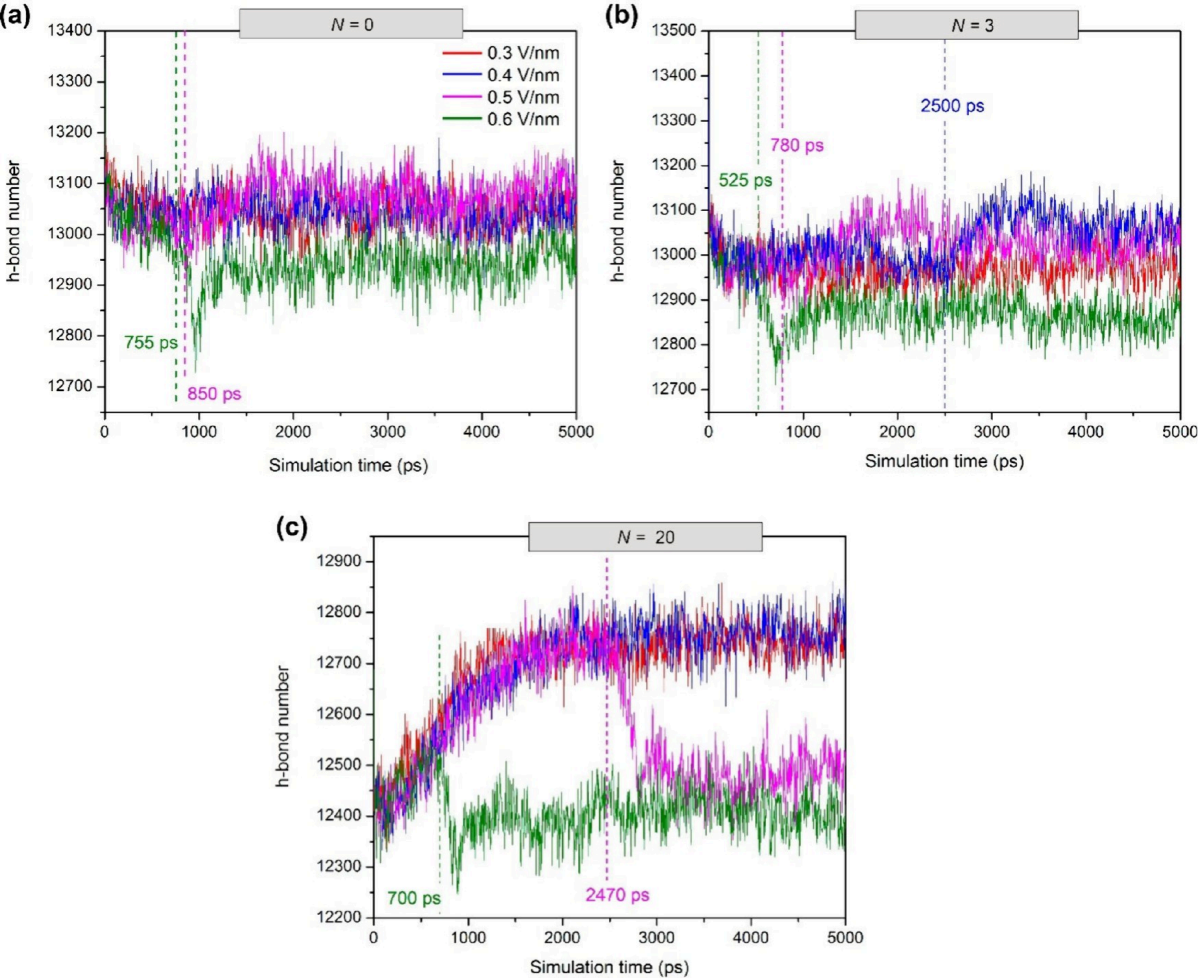
Numbers of hydrogen bonds in water droplets with simulation time for W/O emulsions at different electric field strengths (*E* = 0.3–0.6 V/nm) and numbers of anionic asphaltene molecules per water droplet (*N* = 0, 3, and 20). The dashed vertical lines indicate the onset time (*t*_c_) of water droplet coalescence. The values of *t*_c_ match those obtained by the SASA profiles (Figure 9). The results shown correspond to a replication of the simulation experiments.

**Figure 11:**
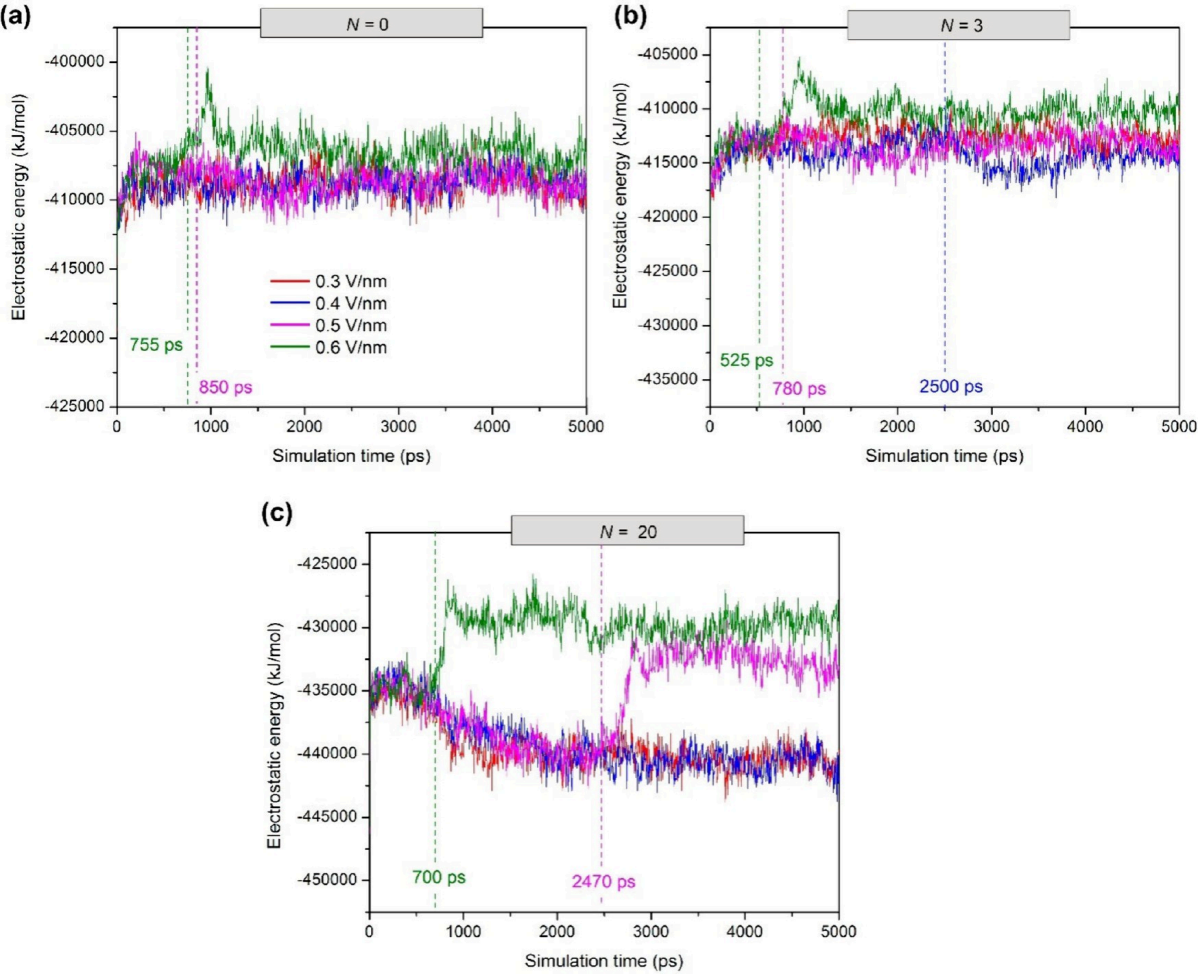
Electrostatic energy in the water droplets with simulation time for W/O emulsions at different electric field strengths (*E* = 0.3–0.6 V/nm) and the number of anionic asphaltene molecules per water droplet (*N* = 0, 3, and 20). The dashed vertical lines indicate the onset time (*t*_c_) of water droplet coalescence. The results shown correspond to a replication of the simulation experiments.

Interestingly, a drastic increase in the number of H-bonds is also observed for processes in which there is no coalescence (see the blue and red curves in Figure 10c before 2470 ps). For these systems at 0.3 and 0.4 V/nm with *N* = 20, there is not enough electrostatic attraction between water molecules of different droplets to cause coalescence. Actually, the increase in the number of hydrogen bonds with simulation time is caused by the migration of anionic asphaltene molecules from the interior of the droplet toward the surface under the application of a strong electric field, thus contributing to strengthening of the hydrogen bond interaction network inside the water droplets and consequently decreasing the SASA, as shown in Figure 9c. Such migration is not observed during the *NPT* equilibration before application of the electric field (section 3.2), revealing that asphaltene aggregation phenomena within water droplets may impair the diffusion of large aggregated molecules toward the water droplet surface. In fact, the strong H-bonds between water molecules and asphaltenes may provide an additional aggregation mechanism for asphaltenes within water droplets,^83,84^ which are broken when a strong electric field is applied, thus assisting the migration of “individual” asphaltenes toward the water droplet surface.

The migration effect of anionic asphaltene molecules and subsequent accumulation on the surface of water droplets reveal the nature of the interfacial activity of this type of asphaltene in the electro-coalescence of W/O droplets. As described previously,^18,34–37^ the anionic asphaltenes at high concentrations on the surface of water droplets can interact between them through π–π stacking, hydrogen bonds, and other noncovalent bonds on the oil–water surface to form aggregates and a viscoelastic interfacial film impeding coalescence,^22^ especially at low *E* values. Even at low *N* contents on the surface of water droplets, where there is virtually no π–π stacking interaction between the aromatic rings of asphaltenes, the asphaltenes interact via hydrogen bonds of their carboxylate groups with the hydrogen atom of water, thus anchoring themselves on the surface of the droplets,^39^ as shown by the radial distribution functions in Figure S3. These very strong interactions keep asphaltene molecules irreversibly adsorbed at the oil–water interface.^160,161^ Indeed, anionic asphaltenes are more readily adsorbed onto the water–oil interface than neutral asphaltenes.^25^

On the other hand, as shown in the snapshots of SASA profiles (Figure 9c), the highest values of *N* (=20) and *E* (0.5 and 0.6 V/nm) facilitate WCC formation between electrodes with a brief initial contact between droplets. Here, unlike coalescence or noncoalescence processes, the formation of a WCC results in water droplet chains between electrodes whose interaction by H-bonds is weak^52^ (Figure 10c) and a high electrostatic energy (Figure 11c). The decrease in the number of H-bonds within the WCC is attributed to the greater separation between the H-bond donor and H-bond acceptor atoms of water molecules, as shown by the highest SASA values in Figure 9c. Because the application of an electric field separates the atoms involved in the hydrogen bonding network of water, there is an increase in surface energy due to the increase in the surface area to volume ratio, and therefore, the electrostatic attraction between the hydrogen bonds is expected to weaken with a less negative electrostatic potential, as illustrated in the profiles at 0.5 and 0.6 V/nm in Figure 11c.

From a molecular point of view, the negatively charged asphaltene molecules are anchored at the water interface by hydrogen bonding (Figure S3) and counterbalanced with positive sodium ions within the water droplets. Such a molecular arrangement of asphaltenes populating the oil–water interphase and ions within water droplets allows a high degree of polarization of the droplet pairs, thus easily adopting a dumbbell-shaped structure^49^ with pronounced cones (section 4.2.3) in the liquid bridge due to strong electric fields.

In the next sections, several electrical and geometric factors of colliding droplets that influence the coalescence onset time (*t*_c_) are assessed (cone angle, surface tension, conductivity, and electrostatic forces), which provides insight into the molecular mechanism of coalescence.

#### 4.2.3 Cone Angle of the Liquid Bridge of Merging Water Droplets

Figure 12 shows cone angle 2β of the liquid bridge between colliding droplets for W/O emulsions studied here. Figure 12 shows that complete droplet–droplet coalescence occurs for β values between 39.5° and 43.8°. This range of β is in agreement with that reported (β ≤ 54.71°) for the coalescence of a conducting droplet pair.^49,78,79^ Since asphaltenes are ionic species and accumulate on the surface of water droplets, it is possible that electrostatic effects are more pronounced at the droplet surface (section 4.2.7), leading to a higher critical electrocapillary number ( $1.18\leq \sqrt{\varepsilon_{c}}\leq 1.37$ (Table 2)) and a β larger than 30.8°^60^ for droplet–droplet coalescence to occur. Figure 12 also shows that there is an increase in β and the electrocapillary number (*ε*_c_) (Figure S4) with *E* ^60,70^ and *N*. These physical properties increase with *N* at a given *E* due to the decrease in the γ of water droplets caused by asphaltenes that accumulated at the interface (section 4.2.4) and the increase in their conductivity (section 4.2.5). At a given *E*, the decrease in γ results in hindering or retarding of droplet–droplet coalescence,^162^ especially in systems having a large amount of asphaltenes. Indeed, when *N* increases from 3 to 20, *ε*_c_ increases (Figure S4) as the γ of water droplets decreases leading to a larger β cone angle and, hence, a longer coalescence time (*t*_c_) according to the following scaling equation:^46,69^

15 $$t_{c}\equiv \sqrt{\rho_{\mathrm{av}}{R_{\mathrm{av}}}^{3}/\gamma }$$

where ρ_av_ = (ρ_w_ + ρ_o_)/2 and *R* = 2*r*_1_ *r*_2_/(*r*_1_ + *r*_2_)^69^ are the average mass density of the two droplets and the effective radius of the droplet pairs, respectively. ρ_w_ and ρ_o_ are the densities of water and oil, respectively, and *r*_1_ and *r*_2_ are the radii of colliding droplets.

**Figure 12:**
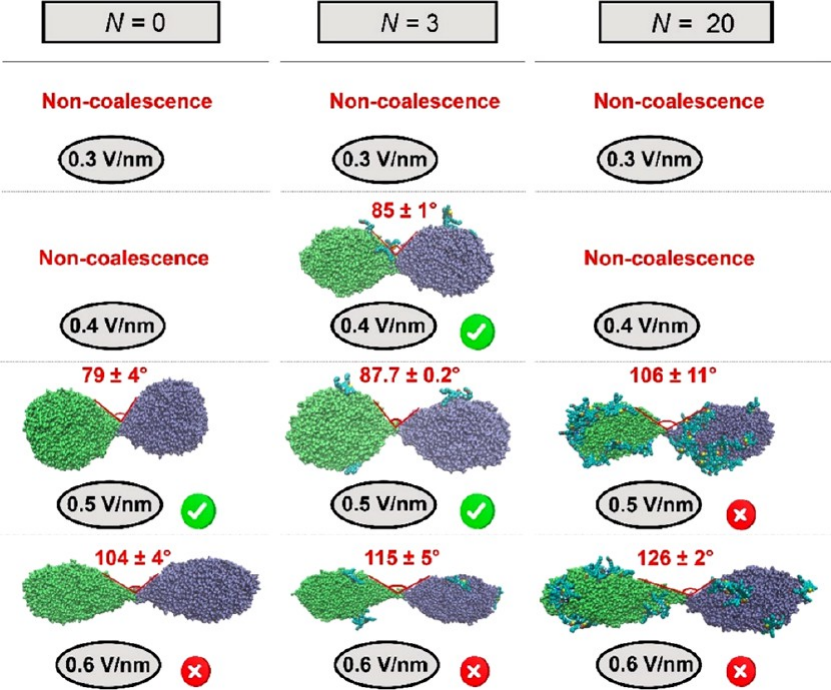
Cone angle 2β of the liquid bridge between colliding droplets in W/O emulsions at different electric field strengths (*E* = 0.3–0.6 V/nm) and the number of anionic asphaltene molecules per water droplet (*N* = 0, 3, and 20). The signs (√ and × ) next to the *E* values indicate whether complete droplet–droplet coalescence has occurred. In the case of water chain configuration (WCC) formation at high *E* and *N* (marked with ×), the colliding water droplets form a large β cone angle during contact but then elongate without achieving complete droplet–droplet coalescence. The results were obtained by averaging three replicates of the simulation experiments.

**Table 2:** Electrocapillarity Number Roots ( $\sqrt{\varepsilon_{c}}$ )­^a^ of Water Droplets at Different Electric Field Strengths (*E* = 0.3–0.6 V/nm) and Numbers of Anionic Asphaltene Molecules per Water Droplet (*N* = 0, 3, and 20) for the W/O Systems Studied^a^

*E*(V/nm)	*N* = 0	*N* = 3	*N* = 20
0.3	ND^b^	ND	ND
0.4	ND	1.31 ± 0.01	ND
0.5	1.18 ± 0.05	1.37 ± 0.002	1.9 ± 0.1
0.6	1.83 ± 0.05	2.24 ± 0.06	2.80 ± 0.02

a The results were obtained by averaging three replicates of simulation experiments.

b The values of the electrocapillary number root ( $\sqrt{\varepsilon_{c}}$ ) were estimated by eq 2 using β values computed in Figure 12.

c ND means not defined because droplet–droplet coalescence was not detected.

However, despite this expected behavior between *t*_c_ and γ, it is possible to observe that when *N* is increased from 0 to 3 at a given *E*, β is augmented (Figure 12), and the systems show a decreased coalescence time (*t*_c_) (Table 1) despite γ being decreased by the addition of asphaltenes to the systems. Here, it is possible that the inner faces of water droplets experience strong Coulombic attraction compared to the rest of the drop and protrude inward to touch and coalesce easily,^16^ which will be demonstrated below (section 4.2.7). On the other hand, from Table 1, it is possible to appreciate that *t*_c_ decreases (thus favoring coalescence) as *E* increases at a given *N* even when the *ε*_c_ (and β (eq 2)) of droplets increases. Since high values of *ε*_c_ have been associated with noncoalescence of droplets, it is reasonable to expect high values of *t*_c_. However, eq 1 shows that the values of *ε*_c_ can also increase with electric field strength (*E*), even when γ increases due to the distribution of asphaltenes at the interface, a hypothesis that will be demonstrated in the next section. Thus, an increase in the γ of droplets should then favor coalescence according to eq 15.

#### 4.2.4 Distribution of Asphaltene Molecules and Na Ions in Water Droplets

The mass density distribution of asphaltenes around the surface of water droplets and sodium cations within droplets is illustrated in Figure 13. Figure 13a shows that for the W/O system with *N* = 3 and *E* ≥ 0.4 V/nm, fewer peaks form, especially for the left droplet that is closer to the positive electrode. This suggests that concentrated monomers exist or aggregates form at specific locations on the surface of water droplets even at low *N* values, which can be confirmed in Figure 14a. In other words, from the snapshot of the motion trajectory for asphaltenes and sodium cations illustrated in this figure, one can see that there is a decrease in the number of asphaltene molecules per water droplet (*N* ≈ 2) in relation to that introduced in the box simulation (*N* = 3) (Figure 14c).

**Figure 13:**
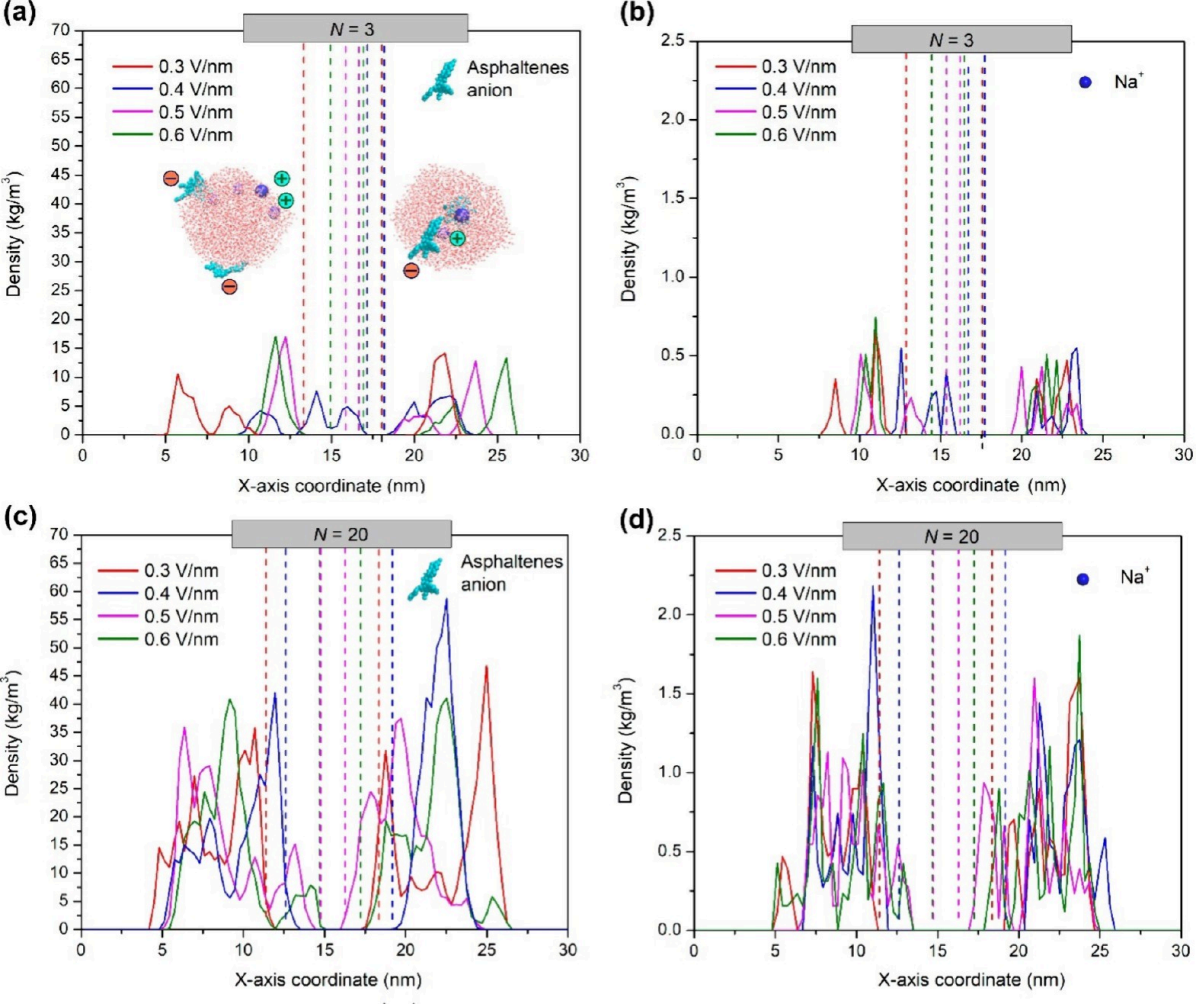
Mass density distribution of (a and c) asphaltenes and (b and d) sodium ions along the *x*-axis coordinate in the water droplets at different electric field strengths (*E* = 0.3–0.6 V/nm) and numbers of anionic asphaltene molecules per water droplet (*N* = 3 and 20). Dashed vertical lines indicate the separation between the faces of water droplets (Figures S5–S7) before the formation of the liquid bridge between them. The results shown correspond to a replication of simulation experiments.

**Figure 14:**
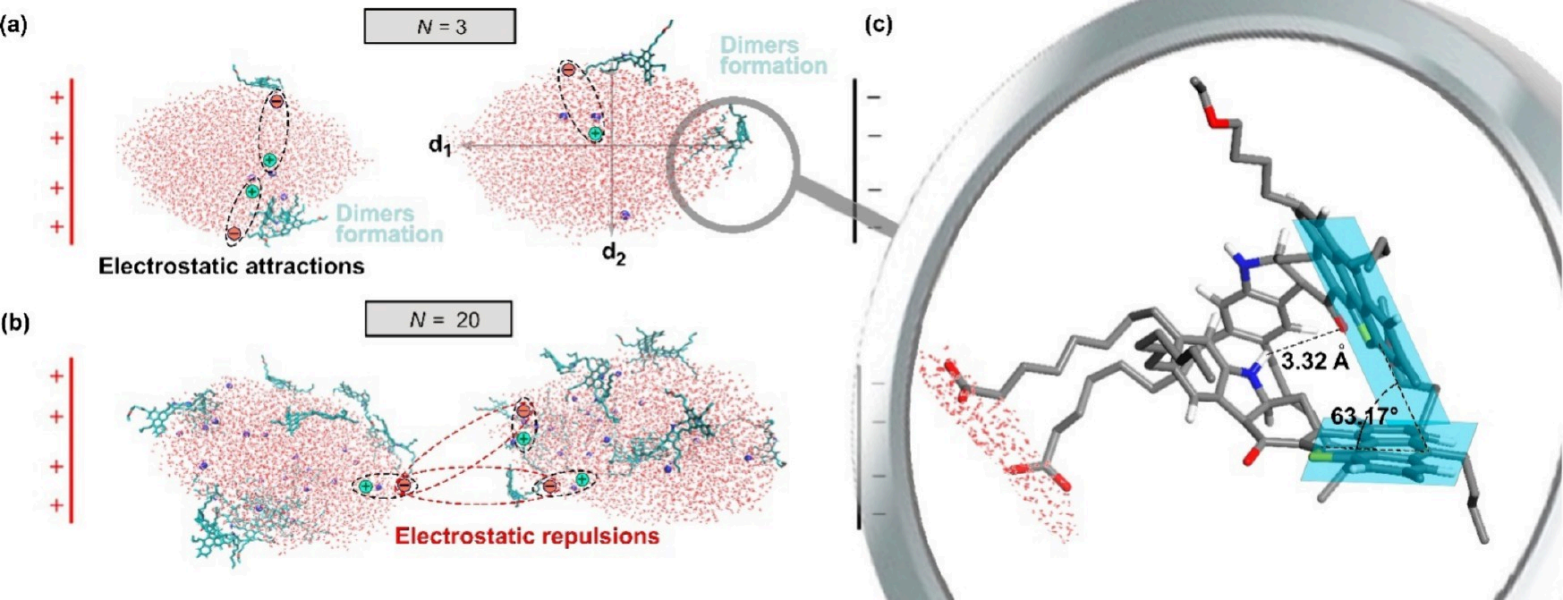
Snapshots of asphaltene molecules and sodium cations at an *E* of 0.6 V/nm and different numbers of anionic asphaltene molecules per water droplet (*N*): (a) *N* = 3 and (b) *N* = 20. The anionic asphaltene molecules are colored cyan, whereas the sodium cations are colored dark blue. The water droplets show oblate spheroids,^165^ where *d*_1_ and *d*_2_ are the semimajor and semiminor axes, respectively. (c) Enlarged view of the asphaltene dimers formed on the water droplet surface for the emulsion system with *N* = 3. An approximately 3.3 Å weak hydrogen bonding interaction is formed between the hydrogen atom (donor) attached to the pyrrole and the carbonyl group (acceptor group) of the conjugate system. This interaction causes π–π T-shaped stacking between asphaltenes and the centroids of interacting aromatic rings,^101^ with an angle between the planes of approximately 63.2°, which is responsible for the aggregation of dimers. A larger amount of asphaltenes (e.g., *N* = 20 (Figure 14b)) allows the formation of asphaltene multimers (dimers and tetramers) on the surfaces of water droplets by face-to-face π–π stacking (Figure S9). The results shown correspond to a replication of simulation experiments.

The above findings suggest that there is slight agglomeration of asphaltenes at low *E* values. On the other hand, upon analysis of the distribution of asphaltenes for the W/O system with *N* = 20 (Figure 13c), the aggregation phenomenon seems to be more evident. The finding of multiple peaks in the distribution profile reveals that aggregate formation rather occurs at different locations on the water droplet surface, as shown in Figure 14b. At high *E* values (above 0.4 V/nm), there are fewer peaks than at lower *E* values (0.3 and 0.4 V/nm), which are more pronounced and located around 5–10 nm on the *x*-axis coordinate for droplet 1. This suggests that, like an increase in *N*, an increase in *E* also causes asphaltene agglomeration at certain locations of droplets and shows clear evidence of the formation of asphaltene aggregates in consonance with electrodeposition experiments.^30^ For the W/O system with *N* = 20, aggregate formation is expected because the number of asphaltene molecules is greater than 6, which is the appropriate number for successful asphaltene aggregation.^93^

According to the calculation of the number of asphaltene molecules at the interface between the inner faces of the water droplets (*N*_asph_^inter^) estimated from the distribution in Figure 13, some free molecules (∼3–5 (Table 3)) of asphaltene can visit the adjacent interface between the two droplets, thereby hindering their coalescence (Video S3). Indeed, at lower values of *E* (0.3 and 0.4 V/nm), where *N*_asph_^inter^ is somewhat small (Table 3) and the polarization of droplets is not sufficient, droplet–droplet coalescence is not observed. Moreover, from the *N*_asph_^inter^ values reported in Table 3, it appears that *N*_asph_^inter^ tends to decrease as *E* increases from 0.5 to 0.6 V/nm. This trend suggests that asphaltene molecules at the interface between water droplets might be agglomerated at other sites on the water surface when strong electric fields are applied. From the interfacial tension (γ) point of view, the convection of these asphaltene molecules from the interface to other sites on the water droplet surfaces would locally increase γ at the interface before creating the liquid bridge, favoring the coalescence and decreasing *t*_c_ with an increase in *E*, as shown in Table 1.

**Table 3:** Numbers of Asphaltene Molecules That Accumulated at the Interface between Droplet Faces (*N*_asph_^inter^) and Interfacial Tension (γ) at Different Electric Field Strengths (*E*) and Numbers of Anionic Asphaltene Molecules per Water Droplet (*N*)­^a^

	*N*_asph_^inter^ ^a^		γ (mN/m)^b^	
*E* (V/nm)	*N* = 3	*N* = 20	*N* = 3	*N* = 20
0.3	0.12 ± 0.06	3.4 ± 0.8	50.5 ± 0.1	44.6 ± 0.9
0.4	0.45 ± 0.03	3.6 ± 0.8	50.2 ± 0.3	47.1 ± 0.1
0.5	0.4 ± 0.1	4.8 ± 0.4	50.5 ± 0.2	48.1 ± 0.3
0.6	0.39 ± 0.09	2.4 ± 0.6	50.6 ± 0.1	49.6 ± 0.8

a The results were obtained by averaging three replicates of simulation experiments.

b *N*_asph_^inter^ was calculated from the integration of the mass density distribution of asphaltenes (Figure 13) along the *x*-axis (Table S5).

c The values of γ and their errors were estimated following the methodology described in Note S2.

Analyzing the density distribution of sodium cations in the water droplets (Figure 13b,d), one can see that all of the peaks corresponding to the cations closest to the surface of the water droplets are far from the boundaries (dashed vertical lines) separating the faces of the water droplets for all systems. Obviously, this reveals that sodium cations do not accumulate on the surface of water droplets but rather within the aqueous phase of the droplets with a certain degree of hydration. The minimum separation between the surface of the water droplet and the sodium cations is equal to 0.5 nm (δ_2_ values in Table S6), corresponding to the system with *E* = 0.5 V/nm and *N* = 20, where there is precisely a large number (∼5) of asphaltene molecules gathered at the adjacent interfaces between water droplets (Table 3). The increased amount of asphaltenes at the interface (especially on droplet 2 (see Table S6)) can be caused by strong electrostatic attractions on the sodium ions contained inside droplets, pushing them toward the surface, as shown in Figure 14b. Here there could be a neutralization of the negative charges of the asphaltene molecules by interfacial sodium cations facilitating the aggregation of asphaltenes between leading edges of water droplets. In this scenario, emulsions with high anionic asphaltene contents, whose water droplets show a decreased surface tension, could give rise to a “gel-like” network structure within a thin oil film, delaying and/or hindering coalescence. This finding is in consonance with some experimental studies,^113,164^ in which salt dissolved in the aqueous phase was found to induce an increase in the stability of W/O crude emulsions favoring the aggregation of neutral asphaltene molecules on water droplets.

This same electrostatic attraction effect between anionic asphaltene molecules and sodium cations might explain why sodium cations are more located at the center of the oblate spheroids of water droplets for the system with *N* = 3. For this system, in general, a larger separation (with a greater δ_2_ (Table S6)) is observed between sodium cations and the surface boundary of droplets, indicating that these cations are rather located at the center of the droplets interacting favorably with closest asphaltene anions that are located on the droplet surface near the semiminor axis (*d*_2_) (Figure 14a). As discussed below, the localization of these cations at the center of the droplets could also favor the polarization of the surface water molecules, decreasing the coalescence time (*t*_c_) for the system with *N* = 3 compared to that with *N* = 0, as shown in Table 1.

So far, the results show that a decrease in γ at the interface between water droplets that provoked by an increase in *N*_asph_^inter^ leads to an increase in *ε*_c_ and β for a given *E* value. This leads to a longer coalescence time for systems with *N* = 20 in relation to those with *N* = 3. However, the decrease in droplet–droplet coalescence time (*t*_c_ (Table 1)) is not explained by the decrease in γ from *N* = 0 to *N* = 3. For example, why, in systems at *E* = 0.5 V/nm with an increase in *N* from 0 to 3, *t*_c_ is reduced even though γ is decreased by the addition of asphaltenes? It is possible that other factors are influencing the electrocapillarity number (*ε*_c_) and β cone angle of the liquid bridge during droplet–droplet coalescence, as species diffusion, conductivity, and polarization of water droplets.^141^ With this objective in mind, we examined the transport and electrical properties of water droplets, which are related to *ε*_c_ and *t*_c_.

#### 4.2.5 Ion’s Diffusion and Conductivity of Water Droplets

Figure 15a displays the self-diffusion coefficient of asphaltene molecules (*D*_asph_) in the water droplets with *N* and *E* for the systems studied here. In this figure, for the system with *N* = 3, the values of *D*_asph_ are higher than those for the system with *N* = 20. As mentioned above (section 4.2.4), this is due to the fact that the molecular structure of asphaltene aggregates in the W/O system with *N* = 3 is rather in a monomeric state^137^ compared to that with *N* = 20, which allows easier translational movement of asphaltene molecules in the first system, and therefore a higher *D*_asph_. With a low number of asphaltenes per water droplet (*N* < 6), asphaltene aggregates have some difficulty forming on the surface of water droplets. However, as one can see from Figure 15a, there is also a decrease in *D*_asph_ starting from *E* ≥ 0.4 V/nm for the system with *N* = 3, which suggests that one cannot rule out the formation of quite small aggregates (from two to three monomers) when a strong electric field is applied. As mentioned above, one can find aggregates forming dimers (*N* ≈ 2).^33^ Both studied W/O systems show a decrease in *D*_asph_ when *E* is increased from 0.4 to 0.6 V/nm, clearly revealing a self-aggregation phenomenon of asphaltenes under strong electric field intensities. In fact, when aggregation occurs, the restriction of the motion of the aggregates on the surface of the water droplets is accompanied by strong attractive interactions with sodium cations inside the water droplets. Therefore, the translational motion of some sodium cations, especially those closer to the interface, is also restricted due to these attractive interactions with the anionic asphaltenes on the surface (Figure 14b). This fact implies that the self-diffusion coefficients of sodium cations (*D*_Na_) (Figure S8) and asphaltenes (*D*_asph_) (Figure 15a) are both decreased, which is more evident under conditions of high *E* (from 0.5 to 0.6 V/nm), where ion pairs may form. Comparing the self-diffusion coefficients of sodium cations (*D*_Na_) and asphaltenes (*D*_asph_) for these systems (Figure 15a and Figure S8), it appears that there is a positive correlation between them. In other words, an increase in the cation’s self-diffusion coefficient leads to an increase in the anionic asphaltene self-diffusion coefficient, which is most noticeable for the system with *N* = 20. Regarding the differences in *D*_Na_ between systems with *N* = 3 and *N* = 20, the greater diffusivities of cations in system with a low ionic concentration (e.g., *N* = 3) originate from less particle–particle interaction.^157^ With a decrease in the cation concentration, the particle interaction is diminished via the hydration and ion pair, thus increasing the cation’s diffusivity of ions compared to systems with high ionic concentrations, as shown in Figure S8.

**Figure 15:**
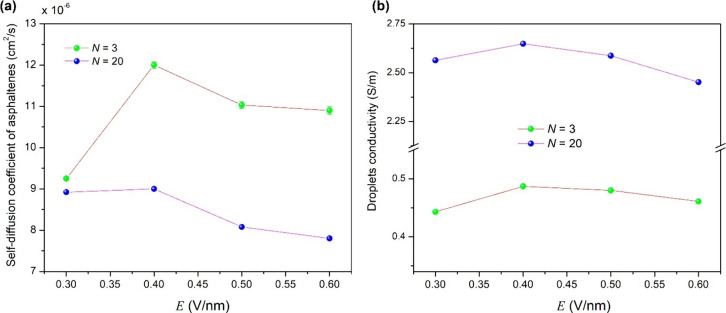
(a) Self-diffusion coefficients of asphaltene molecules around the surface of water droplets (*D*_asph_) and (b) water droplet conductivities (σ) at different electric field strengths (*E* = 0.3–0.6 V/nm) and numbers of anionic asphaltene molecules per water droplet (*N* = 3 and 20). The values of *D*_asph_ at 0.3 V/nm (∼9 × 10^–6^ cm^2^/s) for different values of *N* agree with those obtained by fluorescence correlation spectroscopy at room temperature (3.5 × 10^–6^ cm^2^/s)^137^ and molecular simulation of asphaltenes on Al_2_O_3_ (7.05 × 10^–6^ cm^2^/s).^166^ Self-diffusion coefficient values for species participating in electro-coalescence and droplet liquid conductivity are shown in Table S7.

The total conductivities (σ) of water droplets at different values of *E* for W/O systems are calculated from diffusion coefficients (section 4.2.6) and are shown in Figure 15b. This figure shows that σ values are much higher for the system with *N* = 20 than for the system with *N* = 3 at the same *E*, which is physically reasonable based on a greater number of electrical charges for the first system. For that reason, augmented β cone angles are found as σ (or *N*) increases (Figure 12). The increase in σ should lead to an increase in the meniscus radius of the liquid bridge (*r*_m_),^71^ with Δ*p* decreasing to negative values, thus increasing β values (eq 3). Therefore, it is expected that the increased deformation (due to the high *ε*_c_) with a decrease in γ does not assist the coalescence, as observed at longer coalescence times for a system with *N* = 20 (e.g., 2970 ps) compared to that with *N* = 3 (902 ps) at *E* = 0.5 V/nm (Table 1). This finding explains why the droplet–droplet coalescence time (*t*_c_ (Table 1)) increases when high concentrations of anionic asphaltenes are used with an increased electric force field strength. It is worth mentioning that despite there being formation of some ionic pairs (∼3–5) between sodium cations and anionic asphaltenes at the interface between droplets, the increase in σ of droplets with *N* is not affected, but it does decrease the interfacial tension of water droplets.

Analyzing the behavior of the σ profile with *E* for both systems (Figure 15b), one can see that there is first a slight increase in σ from 0.3 to 0.4 V/nm, which can be attributed to the increase in *D*_asph_ (Figure 15a) by better ion transport with an increase in *E*. On the contrary, at 0.3 V/nm the asphaltene molecules have similar diffusion regardless of *N*; at *E* = 0.4 V/nm, the diffusivity of asphaltene molecules increases, reaching the maximum self-diffusion coefficient (*D*_asph_) and, therefore, the highest σ of droplets. However, at *E* ∼ 0.5 V/nm when there is some degree of asphaltene association (Figure 14b), the diffusivities of both cations and asphaltenes are reduced, resulting in a decrease in the σ of droplets. Because of the attractive interactions between anionic asphaltenes and sodium cations, some ion pairs are formed at the interface between droplets. This decreases the mobility and diffusion of aggregates slowing and/or hindering the electro-coalescence.^157^ Interestingly, the finding of a decrease in σ for the system with *N* = 20 when applying *E* from 0.5 to 0.6 V/nm might indicate that there should be a decrease in the β cone angle of the liquid bridge with a decrease in *r*_m_,^71,72^ which is the opposite result to that found in our work. Instead, we observe a monotonic increase in the β cone angle as *E* increases (Figure 12), which suggests that the behavior of the β cone angle is rather determined by the higher voltage (or *E*) between electrode plates leading to greater deformation (due to the high *ε*_c_) of the droplets. In spite of that, at a given *N*, the coalescence time (*t*_c_) decreases with an increase in electrocapillary number (*ε*_c_), which is in line with reported experimental studies.^4^ In addition to this effect derived from the potential between electrodes, the decrease in *t*_c_ is also promoted by a decrease in asphaltenes at the interface between the two drops of water (*N*_asph_^inter^ (Table 3)), as mentioned above.

#### 4.2.6 Water Diffusivity within Droplets

The difference in the *t*_c_ values for asphaltene-laden water droplet systems has been well explained by considering *N* and *E*. However, the reduction in *t*_c_ for the W/O system with *N* = 3 compared to clean water droplets (*N* = 0) (Table 1) was not clarified. In this context, the behavior of the self-diffusion coefficient of water molecules (*D*_water_) and polarization of water droplets must be analyzed.^157^ Figure 16 shows the values of *D*_water_ for the systems studied here at different values of *E*.

**Figure 16:**
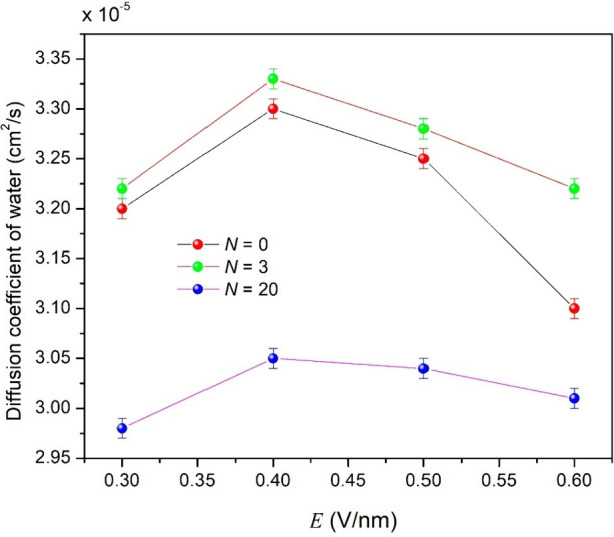
Self-diffusion coefficients of water molecules (*D*_water_) at different electric field strengths (*E* = 0.3–0.6 V/nm) and numbers of anionic asphaltene molecules per water droplet (*N* = 3 and 20). The values of *D*_water_ are in a good agreement with that found experimentally ((2.57 ± 0.02) × 10^–5^ cm^2^/s).^167^

At first glance, Figure 16 shows that there is an increase in *D*_water_ with an increase in *N* from 0 to 3 and then a decrease in *D*_water_ with a further increase in *N* from 3 to 20 for any value of *E*. From these results, we concluded that at a low ionic concentration (e.g., *N* = 3), the diffusivity of water molecules is favored and hence reduced *t*_c_ (Table 1). It is possible that the increase in *D*_water_ is accompanied by an increase of the water molecules’ polarization with ions present inside water droplets.^168^ As described in a prior study,^157^ a low ionic concentration rapidly drives ions toward the water cluster and forms the dipole polarization of the droplet. Besides, we believe that molecular self-diffusivity of water molecules is increased with an increase in electric field intensity due to translational coupling to directly induced rotational motion and the rearrangement of the fields of H-bond networks.^169^ Because of the acceleration in hydrogen bonding kinetics afforded by field-imparted rotational motion, liquid water under an increasing static electric field, in a region close to ambient, has been found to decrease the self-diffusion activation energy and increase *D*_water_.^170^ The greater diffusivity of polarized superficial water molecules of droplets would enhance the drainage and breakage of the thin film and favor the formation of the liquid bridge between droplets with a consequent decrease in *t*_c_.

On the other hand, the diminution of *D*_water_ at a high ionic concentration (e.g., as observed when increasing from *N* = 3 to *N* = 20 (Figure 16)) can be attributed to the enhanced particle–particle interactions. *D*_water_ decreases monotonously with an increase in ion concentration because the ionic hydration effect dominates and the interaction between the hydration shells of cations reduces the mobility of water molecules.^171^ The mobility of both free water molecules and sodium cations is curbed by hydration and then slows the electro-coalescence. The minimum *t*_c_ (and maximum in *D*_water_) at a low ionic concentration and then an increase in *t*_c_ (with a decrease in *D*_water_) at a high ion concentration have been found in experimental studies.^172^ Here, it was found that the efficiency of the electro-coalescence increases first with the increase in ion concentration and then decreases.^172^

To corroborate whether water droplet polarization is a determining factor in droplet coalescence, especially at low ionic concentrations, electric charges, dipole moment, and dipole–dipole forces are investigated in section 4.2.7. As mentioned above, dipole–dipole forces can determine the fate of coalescence, especially when droplets are not rich in asphaltene molecules and their aggregation phenomenon is negligible.

#### 4.2.7 Electrical Charge in Dipole Coalescence

The charge density distribution (ρ­(*x*)) of each droplet before contact was counted just before the formation of the liquid bridge. The profiles of ρ­(*x*) versus the *x*-axis coordinate for each water droplet at different values of *E* and *N* are illustrated in Figures S5–S7. This distribution is due to the electric field-induced polarization and ionic polarization of the oxygen and hydrogen atoms in the water droplet, resulting in an uneven charge distribution in the droplet.^97,173^ In other words, the electrical charge of water droplets is a result of the acquisition of charge by surface water molecules by the polarization of the electric field and of electrical charge induced by dipole polarization of ions (anionic asphaltenes and sodium cations) in the droplet, or so-called ion–ion and ion–surface-induced charge interactions.^174^ To examine how the electrical charge induced by polarization affects the coalescence of water droplets at a given *E*, we used the W/O systems at *E* = 0.5 V/nm with varying *N* values as typical cases. Figure 17 displays the values of ρ­(*x*) for W/O emulsions with *N* = 0 and 3 in which complete droplet–droplet coalescence occurs and with *N* = 20 in which a WCC forms.

**Figure 17:**
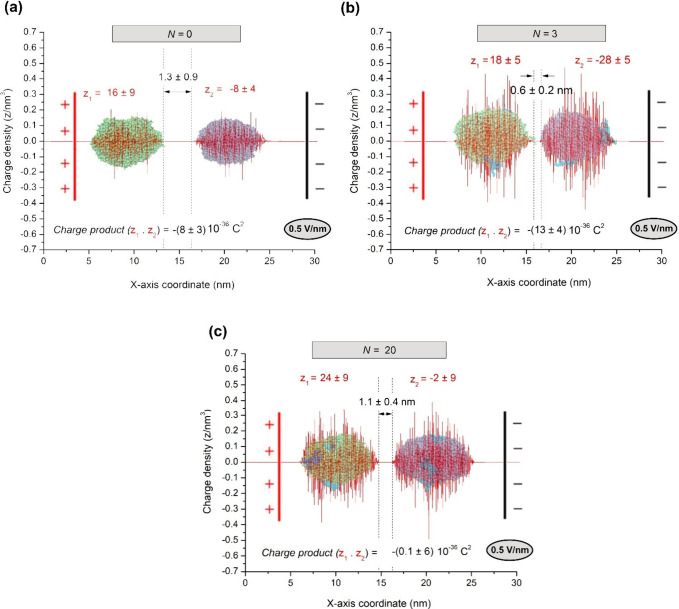
Distribution of the charge density (in solid red lines) for W/O emulsions at *E* = 0.5 V/nm applied along the *x*-axis in the simulation box. The electrical charge of each droplet (*z*_1_ and *z*_2_) before forming a liquid bridge in these emulsions was estimated by eq 8. The distance found by *NPT* simulations between the leading edges of droplets is represented between arrows. The electrical charge product (*z*_1_ *z*_2_ in coulombs) is shown for each system. The geometric mean electrical charge at the interface was estimated by $\sqrt{\left|z_{1}z_{2}\right|}$ , which ranges from 1.2 to 4.1 × 10^–18^ C and agrees with the computed saturation charge (*q*_s__,*x*_, 4.3–8.6 × 10^–18^ C) of a conductive particle of radius *r*_*x*_ with an electrode.^175^ The sodium cations contained in the droplets and *n*-hexane molecules in the W/O systems are not depicted for the sake of clarity.

This figure shows that the systems show opposite electric charges (*z*_1_ and *z*_2_) at each leading edge of the droplets, revealing that the interaction between them is attractive in nature and could bring them closer. Indeed, of all of these systems, the W/O emulsion with *N* = 3 at *E* = 0.5 V/nm exhibits the highest charge modules (|*z*_1_| and |*z*_2_|) for each droplet (Figures S5–S7), and the most negative electric charge product (*z*_1_ *z*_2_ = −13 × 10^–36^ C^2^). In addition, this system shows a fairly short distance between the charged faces of the droplets (0.6 nm (Figure 17b)), thus demonstrating strong dipole forces between droplets during coalescence. Around this distance (∼1.1 nm), the total polarization of the droplet is 80% due to charge interactions induced by sodium cations.^174^ It is possible that a low concentration of monovalent ions (such as Na^+^) increases the polarization of water molecules from the first cluster of hydration shells of cation boosting the dipole moment (0.24–0.4 D by hydrogen bonding) toward the subsequent hydration shells via hydrogen bonding.^168^ The net result of dipolar reinforcement by H-bond-assisted hydration layers would be increased polarization of the molecules at the water surface, which is magnified if the cations are located rather at the center of the droplet due to the greater number in the layers from the center to the surface of the droplets, as shown in the system with *N* = 3 (Figure 14a). Besides this effect, due to the low ionic concentration inside the droplets for the system with *N* = 3, there is a large separation between ion charges, which causes high dipole moments (Table S8) and large polarization of the droplets. This fact would increase the dipole–dipole forces (*F*_dip_) between water droplets at moderate values of *E* (0.4–0.5 V/nm) as shown in Figure 18. Due to this polarization effect opposing the applied electric field (“screening phenomenon”), the electric field within the water droplets is expected to be effectively reduced.^140^

**Figure 18:**
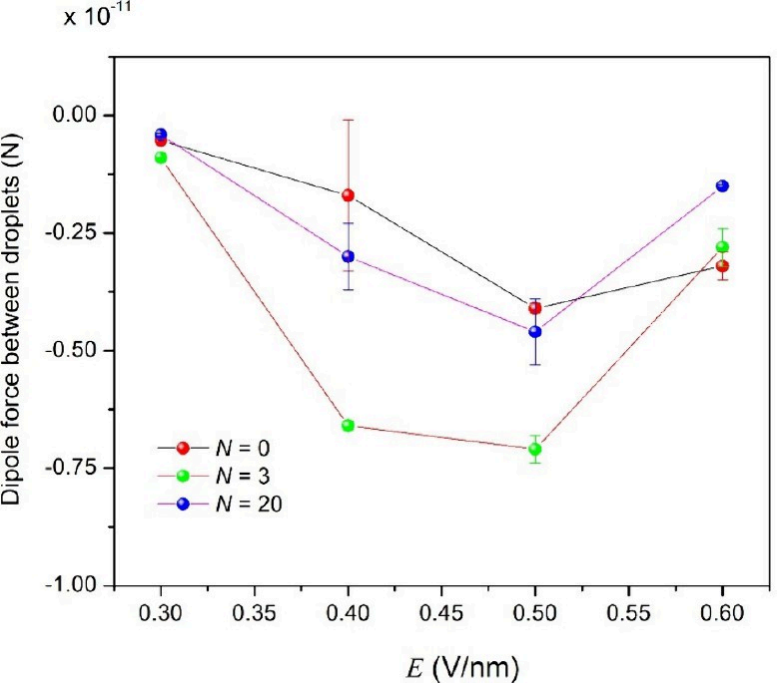
Dipole force (*F*_dip_ (eq 13)) between droplet faces before the formation of a liquid bridge for the W/O systems studied at different anionic asphaltene concentrations: 0–20 asphaltenes per droplet and electric field strength (*E*) of 0.3–0.6 V/nm. The *F*_dip_ values are 6–7 orders of magnitude higher than in experiments (Note S3),^176^ because *E* values used in our MD simulations are approximately 1000 times higher than those used in the experimental design (see Figure 4). The values of *F*_dip_ were obtained by averaging three replicates of the simulation experiments. The error bars were calculated using the formulation of error for *F*_dip_ according to propagation error theory (Note S4).

On the contrary, for the system with *N* = 20, this polarization phenomenon is less pronounced because there are many cations located at the surface (with few hydration water shells) of droplets neutralizing the negatives charges of surface asphaltenes (Figure 14b). This would result in an increase in the positive charge, especially of droplet 2 (*z*_2_) (see Figure 17c), which precipitates a short contact between water droplets without any coalescence and then leads to the formation of a WCC (Video S4). As discussed in section 4.2.4, when *E* is around 0.5 V/nm and there are many molecules of anionic asphaltenes (∼5 (Table 3)) at the interface between water droplets, the high neutralization of these anions with sodium cations results in a larger positive charge of droplet 2 (*z*_2_ = −2 ± 9*e* (Figure S7c)). For that reason, under strong electric fields (*E* > 0.5 V/nm) are found weaker dipolar forces (with more positive values) for asphaltene-laden droplets (systems with *N* = 3 and 20) than for clean water droplets (*N* = 0), as illustrated in Figure 18 (Table S9).

#### 4.2.8 Dipole Force in Dipole Coalescence

Moreover, Figure 18 shows that the dipole force (*F*_dip_) at *E* = 0.3 V/nm is practically zero for all studied systems, which indicates that the polarization of the droplets is not favorable to cause movements in opposite directions to allow them to coalesce, as shown in Figures 9 and 12. However, at *E* values between 0.4 and 0.5 V/nm, a high value of *F*_dip_ (from −0.66 × 10^–11^ to −0.71 × 10^–11^ N) is detected for the system with the lowest number of asphaltenes per water droplet (*N* = 3), revealing that with this dipole force complete droplet–droplet coalescence can be found (Figure 12). As mentioned above, under these conditions of coalescence, there is a high polarization of surface water molecules provoked by hydration of central sodium cations and boosted by hydrogen bonding of water and greater separation between ions in the droplets. Due to these effects, we observe a greater dipolar force (−0.71 × 10^–11^ N) for the system with *N* = 3 than that with *N* = 0 (−0.41 × 10^–11^ N) at *E* = 0.5 V/nm, which implies a shorter coalescence time (*t*_c_ = 902 ps) for the first system than for the latter system (*t*_c_ = 1477 ps). Also, the decrease in *t*_c_ from 1560 to 902 ps in the system with *N* = 3 (Table 1), with an increase in *E* from 0.4 to 0.5 V/nm, is also explained by the slight increase in dipole force between droplets (Figure 18).

Interestingly, from Figure 18, the system with *N* = 20 at 0.5 V/nm has a *F*_dip_ somewhat greater (−0.46 × 10^–11^ N) than that with *N* = 0 (−0.41 × 10^–11^ N), but there is no coalescence in the first system. The droplets touch each other as if they were going to coalesce (Video S4), but due to the high surface electrical charge, they are deformed at this *E* and give rise to water chains (WCC) between the electrodes. A possible reason for this finding is that the coalescence of a system with a high content of asphaltenes (e.g., *N* = 20) is delayed and/or hindered by asphaltene aggregates between leading edges of water droplets (Table 3), despite the high polarization of water droplets. Here, the steric effect predominates over the electrical effect and is more predominant when there is a higher concentration of asphaltenes in the water droplets. At *E* values higher than 0.5 V/nm (e.g., 0.6 V/nm), where the deformation of water droplets and their electrocapillary number increase substantially with a decrease in interface tension and an increase in *E*, a WCC forms in all cases. Under high *E* and *N* values, the value of *F*_dip_ increases to more positive values, which indicates weaker dipole forces at a high electric field intensity. As mentioned above, the enhanced aggregation of asphaltenes at a high *E* is assisted by a neutralization of their negative charges, with the consequent increase in the positive potential of water droplets causing WCC formation.

Finally, all of our results seem to indicate that when *N* is somewhat low (=3) the electrostatic attraction effect between water droplets becomes more important than the self-aggregation effect of asphaltenes on the adjacent interfaces of two droplets, thus enhancing the film thinning/drainage and decreasing *t*_c_. On the contrary, when *N* is very high (=20), the opposite effect is found; in other words, the effect of aggregation and steric hindrance limits the film thinning and outweighs the effect of electrostatic attraction.

## 5 Conclusions

The coalescence of two aqueous water droplets with different anionic asphaltenes placed inside each water droplet (*N*) suspended in an insulating oil and subjected to a DC electric field was studied by molecular dynamics. This study demonstrates that anionic asphaltenes, despite possessing polar groups and electric charge, do not remain in the aqueous medium inside the water droplets; their natural state is to remain at the oil–water interface. The condensed aromatic rings of asphaltene were directed toward the oil phase, while the asphaltene was anchored to the water droplet surface by the interaction of hydrogen bonds between negative carboxylate groups and water molecules. Three behaviors are observed during the coalescence of water droplets loaded with anionic asphaltenes on their surface: (1) noncoalescence, (2) complete coalescence, and (3) formation of a WCC between electrodes. Whereas the poor polarization of water determines noncoalescence, complete droplet–droplet coalescence results from the high polarization of water droplets promoted by the application of a moderate electric field and zero or few asphaltenes. Thus, this study reveals that at moderate *E* values (0.4–0.5 V/nm) and with few asphaltenes per droplet (∼3) in W/O emulsions, there is an optimal condition that favors droplet–droplet coalescence, which may seem counterintuitive when compared to coalescence results in clean water droplets. In this scenario, the few hydrated cations rather located at the center of droplets boost the polarization of interfacial water molecules via hydrogen bonding, thus increasing the diffusion of water droplets by translational coupling to directly induced rotational motion. The high polarization of the water droplets leads to the promotion of coalescence with a reduced coalescence time compared to systems containing a large number of asphaltenes (e.g., ∼20) per water droplet. Here, the disfavoring of coalescence, with an increase in coalescence time, in emulsions containing many anionic asphaltene molecules per water droplet is due to the better aggregation of asphaltenes on the adjacent interface between droplets upon application of a strong electric field. Indeed, the neutralization of the negative charges of these asphaltenes on the surface by the sodium cations within the droplets allows the effective formation of a film between the droplets, hindering and/or delaying coalescence. From all of our results, it appears that as *N* increases at a given *E*, the dominant factor is the droplet conductivity (σ) rather than the interface tension (γ) of water droplets. Increasing *N* will increase σ and the electrocapillary number (*ε*_c_), which increases droplet deformation and hinders droplet–droplet coalescence (with a longer coalescence time). On the other hand, when *E* is increased at a given *N*, the γ of water droplets is the predominant factor in their coalescence. In other words, increasing *E* will cause an increase in γ at the interface between water droplets due to the decrease in *N*_asph_^inter^, with a consequent decrease in the coalescence time (*t*_c_). This effect of the interfacial tension cooperates with the higher voltage applied between electrodes to reduce *t*_c_ and favor coalescence. The interplay of factors such as γ, σ, and *E* together with the number of asphaltenes (*N*) must be considered to find “optimal conditions” for electro-coalescence of W/O emulsions containing ionic asphaltenes at a high water pH. From an industrial point of view, it is possible to control the pH of the emulsion to obtain a suitable number of anionic asphaltenes on the water droplet surface, wherein the conductivity (σ) of droplet is relatively low and the interfacial tension (γ) is somewhat high at moderate values of *E* (below the critical *E*), thus favoring complete droplet–droplet coalescence (e.g., with values of $1.18\leq \sqrt{\varepsilon_{c}}\leq 1.37$ ) and avoiding WCC formation, which leads to retarding and/or hindering of coalescence and impairing dewatering of oil.

Despite the limitations and assumptions considered in our MD simulations to reduce the computational time, the results were consistent with other experimental and theoretical findings. Based on some idealized assumptions, this work intended to simulate complex systems involving structurally complex asphaltene molecules at the water–oil interface under an imposed electric field in the presence of ions. As higher computing speeds become available in the future, more realistic attempts to refine the above assumptions (section 3.5) will be possible.

## Supplementary Material











## Notes

Asphaltene density profiles on water droplet surfaces at *N* values starting from different simulation conditions, with asphaltenes placed at the water–oil interface and inside the water droplet; droplet–droplet coalescence onset times at different electric field strengths (E) with three asphaltene molecules per droplet in W/O emulsions when asphaltenes were initially located on the water droplet surface; deformation ratio of each droplet calculated at different electric field strengths and numbers of asphaltenes per droplet in W/O emulsions; calculation of critical thickness of film drainage; values of K estimated by eq 11; products of electrical charges and separation distances between water droplets before the creation of the liquid bridge at different electric field strengths and numbers of asphaltenes per droplet in W/O emulsions; contributions of electrostatic and van der Waals energies to the total potential energy for the W/O systems containing zero asphaltenes per droplet (“clean droplets”) at different electric field strengths; radial distribution function between the oxygen atom of the asphaltene carboxylate group and the hydrogen atom of water for W/O emulsions containing 3 asphaltene molecules per droplet subjected to different electric field intensities; electrocapillarity number of water droplets before the formation of the liquid bridge in W/O emulsions at different electric field strengths and numbers of anionic asphaltene molecules per water droplet; distribution of charge density of two coalescing water droplets with 0, 3, and 20 asphaltene molecules under a DC electric field applied along the *x*-axis of the simulation box; estimation of interfacial tension at the interface between leading edges of water droplets; percents of asphaltenes on the adjacent interface per droplet at different electric field strengths and numbers of asphaltenes per droplet in W/O emulsions; distances of sodium cations from the interior to the surface of the water droplets at different electric field strengths and numbers of asphaltenes per droplet in W/O emulsions; self-diffusion coefficients of sodium cations within water droplets at different electric field strengths and numbers of anionic asphaltene molecules per water droplet; conductivity and self-diffusion coefficient of asphaltenes with sodium cations and water molecules in the droplets at different electric field strengths and numbers of asphaltenes per droplet in W/O emulsions; dipole moment along the *x*-axis of the simulation box at different electric field strengths and numbers of asphaltenes per water droplet in W/O emulsions; estimation of dipole forces by scaling analysis; derivation of the error formulation for dipole forces according to error propagation theory; calculated values of dipole forces between water droplets before the creation of the liquid bridge at different electric field strengths and numbers of asphaltenes per droplet in W/O emulsions; and structures of the multimers (dimer (a) and tetramer (b)) found during droplet–droplet coalescence in the emulsion system with 20 anionic asphaltene molecules under application of an electric field equal to 0.6 V/nm (PDF)
Video S1 (MPG)
Video S2 (MPG)
Video S3 (MPG)
Video S4 (MPG)

## List of Symbols

*h*_c_: critical thickness during film thinning/drainage of the liquid before coalescence (nm)
*r*_m_: meniscus radius of the liquid bridge (nm)
*ε*_c_: electrocapillary number
*t*_c_: coalescence time when two water droplets begin to form the liquid bridge (ps)
ϵ: relative static permittivity of water
ϵ_0_: absolute permittivity of a vacuum (C^2^ N^–1^ m^–2^)
*E*: electric field strength (V/nm)
*a*: radius of a water droplet (nm)
γ: surface/interfacial tension of water droplets (*m*N/m)
β: cone angle of the liquid bridge (deg)
Δ*p*: pressure difference between the bulk of the droplet and the meniscus bridge (Pa)
*p*_droplet_: pressure of the droplet bulk (Pa)
*p*_bridge_: pressure of the meniscus bridge (Pa)
σ: conductivity (S/m)
*g*(*r*): radial pair distribution funtion at a distance *r*
*d*: droplet diameter (nm)
*z*: electrical charge on a certain portion of the droplet’s surface (C)
ρ­(*x*): density distribution of electric charge in the *x* coordinate (*z*/nm^3^)
*N*: number of asphaltenes per droplet water
*F*_dip_: dipole–dipole interaction force beween droplets (N)
ω: Clausius–Mossotti factor
*ϵ*_d_: water dielectric constant (C^2^ N^–1^ m^–2^)
*ϵ*_c_: oil dielectric constant (C^2^ N^–1^ m^–2^)
*ϵ*_m_: medium permittivity (C^2^ N^–1^ m^2^)
*r*_1_: radius of droplet 1 (nm)
*r*_2_: radius of droplet 2 (nm)
*S*: separation distance between the center of the droplets (nm)
*N*_asph_^inter^: number of asphaltenes that accumulated at the interface between the inner faces of the water droplets
*D*_1_: deformation of droplet 1 under electric fields
*D*_2_: deformation of droplet 2 under electric fields
ρ­(*x*): mass density distribution of asphaltenes along the *x*-axis coordinate (kg/m^3^)
*x*: *x*-coordinate (nm)
*D*_*i*_: self-diffusion coefficient for species *i* (cm^2^/s)
*q*_*i*_: charge of species *i* (C)
*C*_*i*_: concentration of species *i* (mol/cm^3^)
*k*_B_: Boltzmann constant (J/K)
*T*: temperature (K)
*r*_*i*_(*t*): position of species *i* at time *t* (nm)
MSD_*i*_: mean square displacement of species *i* (cm^2^)
*d*: distance between leading edges of droplets (nm)
*t*: simulation time (ps)
*d*_1_: semimajor axis of the oblate ellipsoid droplet (nm)
*d*_2_: semiminor axes of the oblate ellipsoid droplet (nm)
SASA: solvent accessible surface area of water droplets (nm^2^)
