# Supplementary material for: Molecular Dynamics Study of the Coalescence of Water Droplets with Anionic Asphaltene Molecules under a DC Electric Field
Source: Langmuir. 2025 Sep 2;41(36):24593–618. doi: 10.1021/acs.langmuir.5c02957 (PMC12445008; doi:10.1021/acs.langmuir.5c02957)
Supplement: Supplementary file 1 [file la5c02957_si_001.pdf]

A Molecular Dynamics Study of the Coalescence of Water Droplets with Anionic Asphaltene Molecules Under DC Electric Field

Jurgen Lange Bregado,*[a] Argimiro R. Secchi,[a,b] Marcio Nele[a,b]

[a] Chemical Engineering Program, COPPE, Universidade Federal do Rio de Janeiro, Cidade Universitária, Rio de Janeiro CP: 21941-914, Brazil.

[b] Chemical and Biochemical Process Engineering Program, Escola de Química, Universidade Federal do Rio de Janeiro, Cidade Universitária, Rio de Janeiro. CP: 21941-909, Brazil.

Corresponding author, email: jurgenlange1967@gmail.com (J. L. B)

This file includes: 26 pages, 9 Tables, 9 Figures, 4 Notes

**Computational methodology**		Page S3-7
**Result details**		Page S8-24
**References**		Page S25-26
**Figure S1**	Asphaltene density profiles on water droplet surfaces at N = 20 and E = 0.6 V/nm starting from different simulation conditions: with asphaltenes placed at the water/oil interface and inside the water droplet	Page S3
**Table S1**	Droplet-droplet coalescence onset time at different electric field strength (E) and (N=3) asphaltene molecules per droplet in W/O emulsions when asphaltenes were initially located on the water droplet surface	Page S4
**Table S2**	Deformation ratio of each droplet and surface area of the spherical caps between the edges of the water droplets at different electric field strength and number of asphaltenes per droplet in W/O emulsions	Page S5
**Note S1**	Calculation of critical thickness of film drainage	Page S6
**Table S3**	Values of K estimated by Eq. 11 in the text	Page S7
**Table S4**	Product of electrical charges and separation distance between water droplets before creating the liquid bridge at different electric field strength and number of asphaltenes per droplet in W/O emulsions	Page S8
**Figure S2**	Contributions of electrostatic and Van der Waals energy to the total potential energy for the W/O systems containing 0 asphaltenes per droplet “clean droplets” at different electric field strengths	Page S9
**Figure S3**	Function of the radial distribution between the oxygen atom of the asphaltene carboxylate group and the hydrogen atom of water for W/O emulsions containing 3 asphaltene molecules per droplet subjected to different electric field intensities	Page S10
**Figure S4**	Electrocapillarity number of water droplets before the formation of liquid bridge in W/O emulsions at different electric field strengths and number of anionic asphaltene molecules per water droplet	Page S11

**Figure S5**	Distribution of charge density of two coalescing water droplets with 0 asphaltene molecules under DC electrical field applied along the x-axis of simulation box	Page S12
**Figure S6**	Distribution of charge density of two coalescing water droplets with 3 asphaltene molecules under DC electrical field applied along the x-axis of simulation box	Page S13
**Figure S7**	Distribution of charge density of two coalescing water droplets with 20 asphaltene molecules under DC electrical field applied along the x-axis of simulation box	Page S14
**Note S2**	Estimation of interfacial tension (γ) at the interface between leading edges of water droplets	Page S15
**Table S5**	Percent of asphaltenes on the adjacent interface per each droplet at different electric field strength and number of asphaltenes per droplet in W/O emulsions	Page S16
**Table S6**	Distances of sodium cations from the interior to the surface of the water droplets at different electric field strength and number of asphaltenes per droplet in W/O emulsions	Page S17
**Figure S8**	Self-diffusion coefficient of sodium cations within water droplets at different electric field strengths and number of anionic asphaltene molecules per water droplet	Page S18
**Table S7**	Conductivity and self-diffusion coefficient of anionic asphaltenes, sodium cations and water molecules in the droplets at different electric field strength and number of asphaltenes per droplet in W/O emulsions	Page S19
**Table S8**	Dipole moment along X-direction of simulation box at different electric field strength and number of asphaltenes per water droplet in W/O emulsions	Page S20
**Note S3**	Estimation of dipole forces by scaling analysis	Page S21
**Note S4**	Derivation of the error formulation for dipoles forces according to errors propagation theory	Page S22
**Table S9**	Calculated values of dipole forces [a)] between water droplets before creating the liquid bridge at different electric field strength (E) and number of asphaltenes per droplet (N) in W/O emulsions	Page S23
**Figure S9**	Structure of the multimers [dimer (a), tetramer (b)] found during droplet-droplet coalescence in the emulsion system with 20 anionic asphaltene molecules under application of an electric field equal to 0.6 V/nm	Page S24

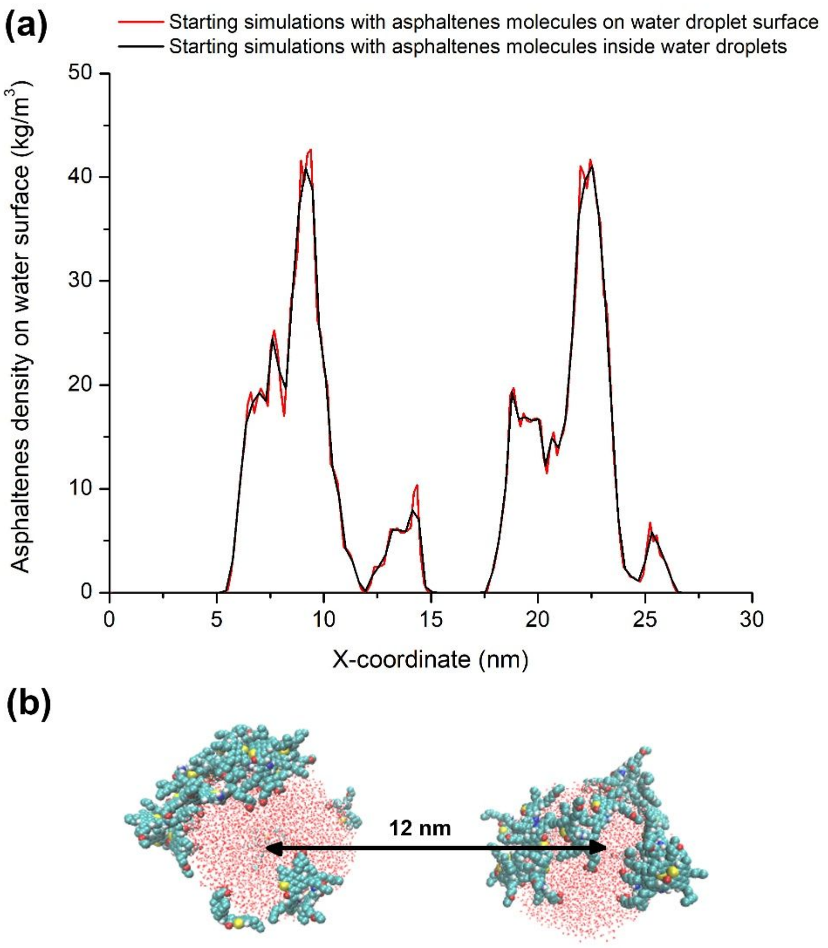

Figure S1a. Asphaltene density profiles on water droplet surfaces at N = 20 and E = 0.6 V/nm starting from different simulation conditions: with asphaltenes initially placed at the water/oil interface (curve in red solid line) (**Figure S1b**) and inside the water droplet (curve in black solid line).

Note: Starting from the initial configurations obtained in this work in which asphaltene molecules are located inside the droplets, these molecules were placed on the surfaces of the water droplets by applying a strong electric field (E = 0.6 V/nm) and constraining the positions of the center of mass of each droplet by non-equilibrium pulling techniques (**Figure S1b**).

Table S1. Droplet-droplet coalescence onset time (t_c, ps) [a)] at different electric field strength (E) and (N=3) asphaltene molecules per droplet in W/O emulsions when asphaltenes were initially located on the water droplet surface.

E (V/nm)	$N = 3$
0.3	ND
0.4	> 5000 ns
0.5	1320
0.6	915

Table S2. Deformation ratio of each droplet (D_1 and D_2) and surface area of the spherical caps between the edges of the water droplets (A_s) calculated at different electric field strength (E in V/nm) and number of asphaltenes per droplet (N) in W/O emulsions

	d_1 [a)]	d_2	$D_1 = \frac{d_1 - d_2}{d_1 + d_2}$	d_1	d_2	$D_2 = \frac{d_1 - d_2}{d_1 + d_2}$	A_s [b)] (nm^2)
E (N)	***Droplet* 1**			***Droplet* 2**			
0.3 (0)	3.5 $\pm$ 0.6	3.2 $\pm$ 0.5	0.04 $\pm$ 0.01	3.7 $\pm$ 0.4	3.2 $\pm$ 0.5	0.05 $\pm$ 0.00	20 $\pm$ 2
0.4 (0)	4.25 $\pm$ 0.05	3.65 $\pm$ 0.05	0.07 $\pm$ 0.01	4.2 $\pm$ 0.0	3.6 $\pm$ 0.1	0.07 $\pm$ 0.01	32 $\pm$ 3
0.5 (0)	4.2 $\pm$ 0.4	2.9 $\pm$ 0.2	0.175 $\pm$ 0.005	4.0 $\pm$ 0.3	3.0 $\pm$ 0.2	0.15 $\pm$ 0.01	73 $\pm$ 2
0.6 (0)	4.5 $\pm$ 0.4	2.9 $\pm$ 0.3	0.21 $\pm$ 0.01	4.9 $\pm$ 0.5	3.1 $\pm$ 0.4	0.22 $\pm$ 0.01	97 $\pm$ 3
0.3 (3)	3.7 $\pm$ 0.7	3.6 $\pm$ 0.7	0.025 $\pm$ 0.005	3.7 $\pm$ 0.5	3.3 $\pm$ 0.4	0.06 $\pm$ 0.01	19 $\pm$ 2
0.4 (3)	3.9 $\pm$ 0.1	3.2 $\pm$ 0.3	0.09 $\pm$ 0.03	3.8 $\pm$ 0.6	3.2 $\pm$ 0.4	0.09 $\pm$ 0.02	41 $\pm$ 8
0.5 (3)	4.6 $\pm$ 0.2	3.35 $\pm$ 0.05	0.15 $\pm$ 0.01	4.35 $\pm$ 0.05	3.3 $\pm$ 0.1	0.14 $\pm$ 0.02	66 $\pm$ 5
0.6 (3)	4.7 $\pm$ 0.7	2.9 $\pm$ 0.3	0.23 $\pm$ 0.02	4.6 $\pm$ 0.0	3.0 $\pm$ 0.2	0.21 $\pm$ 0.03	99 $\pm$ 8
0.3 (20)	3.6 $\pm$ 0.4	3.3 $\pm$ 0.2	0.04 $\pm$ 0.02	3.5 0.1	3.1 $\pm$ 0.2	0.06 $\pm$ 0.02	23 $\pm$ 6
0.4 (20)	4.1 $\pm$ 0.6	3.4 $\pm$ 0.4	0.085 $\pm$ 0.005	4.2 $\pm$ 0.2	3.25 $\pm$ 0.05	0.13 $\pm$ 0.02	49 $\pm$ 5
0.5 (20)	5.1 $\pm$ 0.2	3.2 $\pm$ 0.2	0.23 $\pm$ 0.01	4.45 $\pm$ 0.05	3.1 $\pm$ 0.2	0.17 $\pm$ 0.03	90 $\pm$ 7
0.6 (20)	4.9 $\pm$ 0.5	2.6 $\pm$ 0.1	0.30 $\pm$ 0.03	4.2 $\pm$ 0.5	2.7 $\pm$ 0.5	0.22 $\pm$ 0.03	118 $\pm$ 10

a) d_1 is the end-to-end length of the droplet measured along the axis of symmetry (or semi-major axis, **Figure 14** in the text) and d_2 is the maximum breath in the traverse direction (semi-minor axis, **Figure 14** in the text) of the droplet with oblate spheroid shape.[1,2] The values of d_1 and d_2 correspond to scaled values and not absolute values in nm. Because of d_1 is greater than d_2 ($D_1, D_2 > 0$) in the droplets, they show a geometry of oblate spheroids.[2]

b) Values of A_s were determined by **Eq. S23** in note **S2**

Note S1. Calculation of critical thickness of film drainage

The value of critical thickness (h_c) of film drainage before forming the liquid bridge between colliding water droplets can be estimated as follows:[3,4]

$$h_{\mathrm{c}} = \left(\frac{A_{313}a}{8\pi\gamma}\right)^{1/3} \tag{S11}$$

where A_{313} is the Hamaker constant for two water droplets (component 3) embedded in a n-hexane medium (component 1).

A_{313} can be estimated using the additivity combination rules, where $A_{313} = A_{33} + A_{11} - 2A_{31}$. Here $A_{31} = \sqrt{A_{33}\, A_{11}}$.[6] The values of A_{33} (for water) and A_{11} (n-heptane) were 3.28 10^{-13} and 2.13 10^{-13} ergs, respectively.[5] As an approximation, the value of A_{11} (n-heptane) was considered for n-hexane. Using the additivity combination rules, the computed value of A_{313} was 0.124 10^{-13} ergs (or 0.124 10^{-20} J). Considering that the radius of droplet (a) in our simulation is 3 nm and interfacial tension (γ) of hexane/water is 50.7 mN/m,[6] the value of h_{c} is equal to 0.14 nm. This calculated value is in simulation scale due to the small radius (a) (in nanometric scale) introduced into Equation S11.

Table S3. Values of K [a] estimated by **Eq. 11** in the text

	Asphaltene molecules number per water droplet		
E (V/nm)	$N = 0$	$N = 3$	$N = 20$
0.3	1.050 $\pm$ 0.005	1.03 $\pm$ 0.00	1.04 $\pm$ 0.00
0.4	1.2 $\pm$ 0.1	1.22 $\pm$ 0.02	1.12 $\pm$ 0.05
0.5	1.13 $\pm$ 0.01	1.24 $\pm$ 0.01	1.16 $\pm$ 0.03
0.6	1.07 $\pm$ 0.01	1.070 $\pm$ 0.005	1.05 $\pm$ 0.00

a) The values of S - separation distance between droplet centers - used to calculate K are reported in **Table S4**

Table S4. Product of electrical charges [a] and separation distance (S) [b] between water droplets before creating the liquid bridge at different electric field strength (E) and number of asphaltenes per droplet (N) in W/O emulsions.

E (V/nm)	N		
	0	3	20
0.3	-360 $\pm$ 65 [a]<br>11.4 $\pm$ 0.2 [b]	-165 $\pm$ 96<br>12.7 $\pm$ 0.1	-72 $\pm$ 62<br>12.0 $\pm$ 0.2
0.4	-266 $\pm$ 167<br>9.9 $\pm$ 2.3	-237 $\pm$ 59<br>8.2 $\pm$ 0.1	253 $\pm$ 145<br>9.7 $\pm$ 1.1
0.5	-128 $\pm$ 96<br>9 $\pm$ 1	-504 $\pm$ 110<br>8.1 $\pm$ 0.1	-48 $\pm$ 25<br>8.8 $\pm$ 0.4
0.6	-292 $\pm$ 10<br>10.6 $\pm$ 0.5	-301 $\pm$ 168<br>10.7 $\pm$ 0.3	-460 $\pm$ 166<br>11.25 $\pm$ 0.05

a) Product of electrical charges ($z_1 * z_2$ in e^2) between leading edges of water droplets (**Figure 5** in the text). The electric charges (z_1 and z_2) of each droplet (**Figure S5-S7**) were calculated for a surface layer of excess charge in droplets with a thickness equal to 1.5 nm.[7]

b) Separation distance is defined between droplet centers (S in nm) before creating the liquid bridge between water droplets.

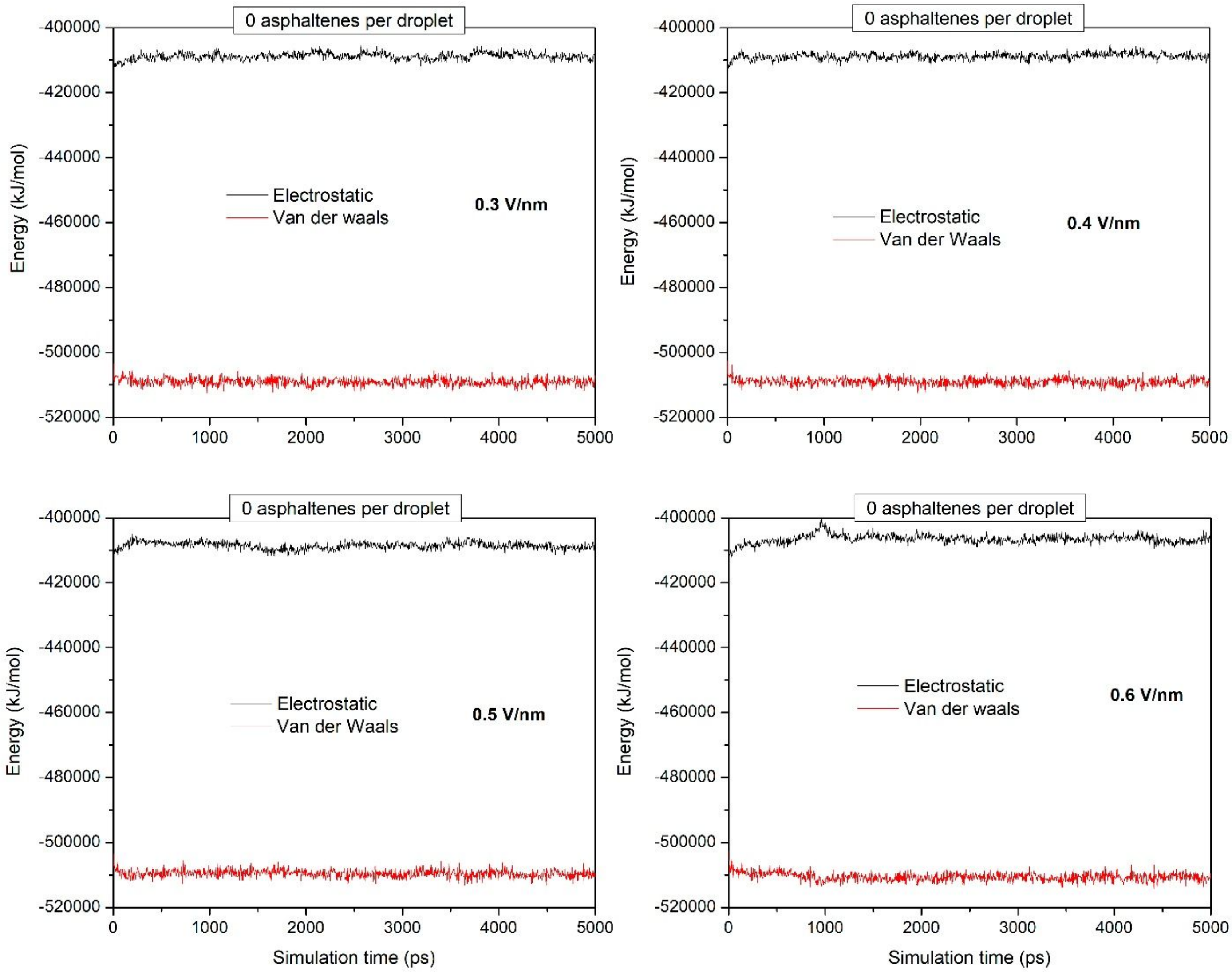

Figure S2. Contributions of electrostatic and Van der Waals energy to the total potential energy for the W/O systems containing 0 asphaltenes per droplet "clean droplets" at different electric field strengths (E): 0.3-0.6 V/nm. The same behavior is observed for the other W/O systems studied.

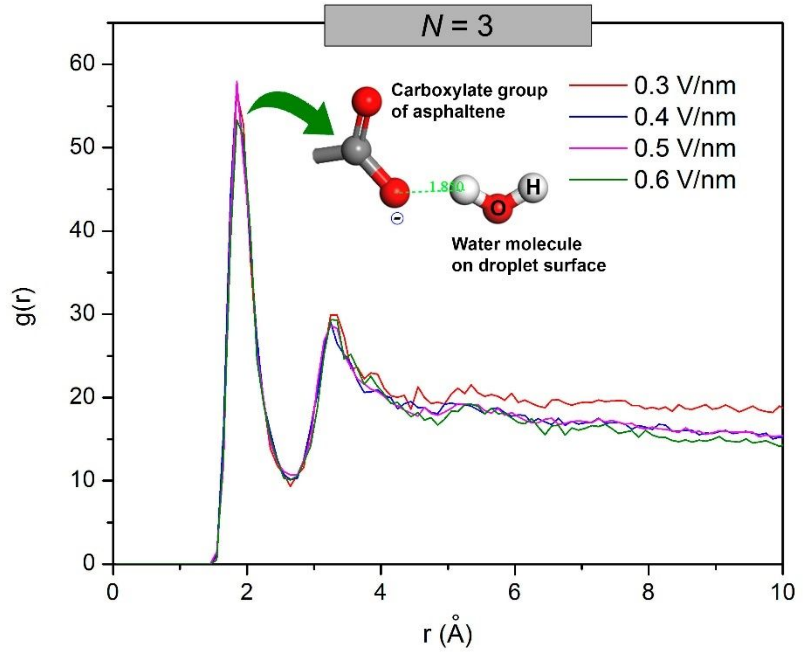

Figure S3. Function of the radial distribution, $\mathrm{g(r)}$ (**Eq. 7** in the text), between the oxygen atom of the asphaltene's carboxylate group and hydrogen atom of water for W/O emulsions containing 3 asphaltene molecules per water droplet (N) at different electric field strength (E): 0, 3 - 0.6 V/nm. Here, it can be observed that there is a maximum corresponding to a distance of 1.85 Å regardless of the electric field strength applied to the emulsions, which reveals that there is a strong interaction of hydrogen bonds between the carboxylate groups of asphaltenes and water molecules on the droplet surface. This type of anionic asphaltene accumulates at the water/oil interface due to this interaction, whereas the nonpolar moiety (aromatic rings) interacts completely with oil.

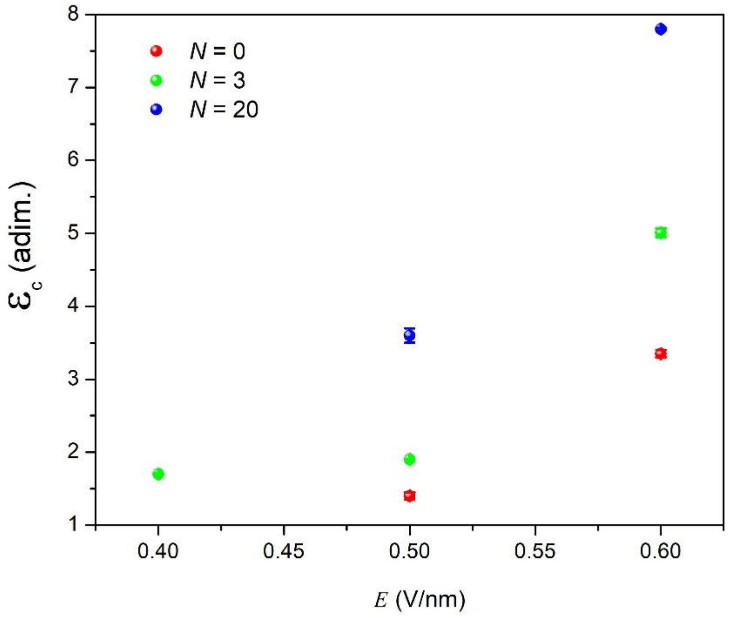

Figure S4. Electrocapillarity number (ε_c) of water droplets before the formation of liquid bridge in W/O emulsions at different electric field strengths (E): 0.3-0.6 V/nm and number of anionic asphaltene molecules per water droplet (N): 0, 3 and 20. The ε_c values are the squares of those listed in **Table 2** in the text. Values are reported where there is complete coalescence or WCC formation (**Figure 12** in the text).

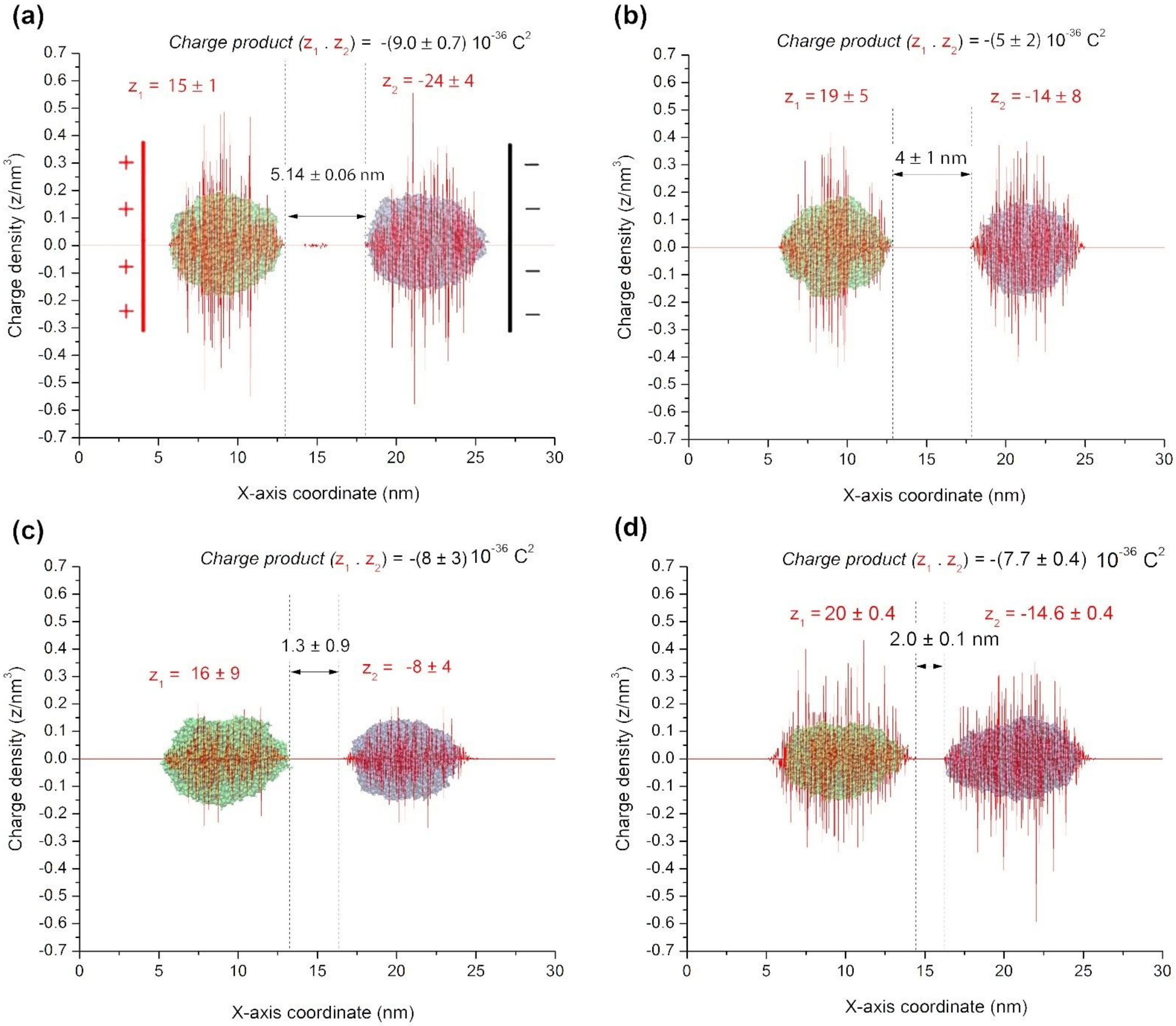

Figure S5. Distribution of charge density ($\rho(x)$ in solid red lines) of two coalescing water droplets with 0 asphaltene molecules under DC electrical field (E): (**a**) 0.3 V/nm, (**b**) 0.4 V/nm, (**c**) 0.5 V/nm, (**d**) 0.6 V/nm applied along the x-axis of simulation box. The electrical charge of each droplet (z_1 and z_2) before forming a liquid-bridge in these emulsions was estimated by **Eq. 8** in the text. The distance (d) found by NPT simulations between leading edges of droplets is represented between arrows. The electrical charge product ($z_1 .$ z_2 in Coulomb units (C) is shown for each system. The geometric mean electrical charge at the interface was estimated by $\sqrt{|z_1 . \ z_2|}$, which ranges from 2.2 to 3.0 x 10^{-18} C and agrees with computed saturation charge ($q_{s,x}$, 4.3 - 8.6 x 10^{-18} C) of a conductive particle of radius r_x with an electrode.[8] The sodium cations contained within the droplets and n-hexane molecules in the W/O systems are not depicted for clarity reasons.

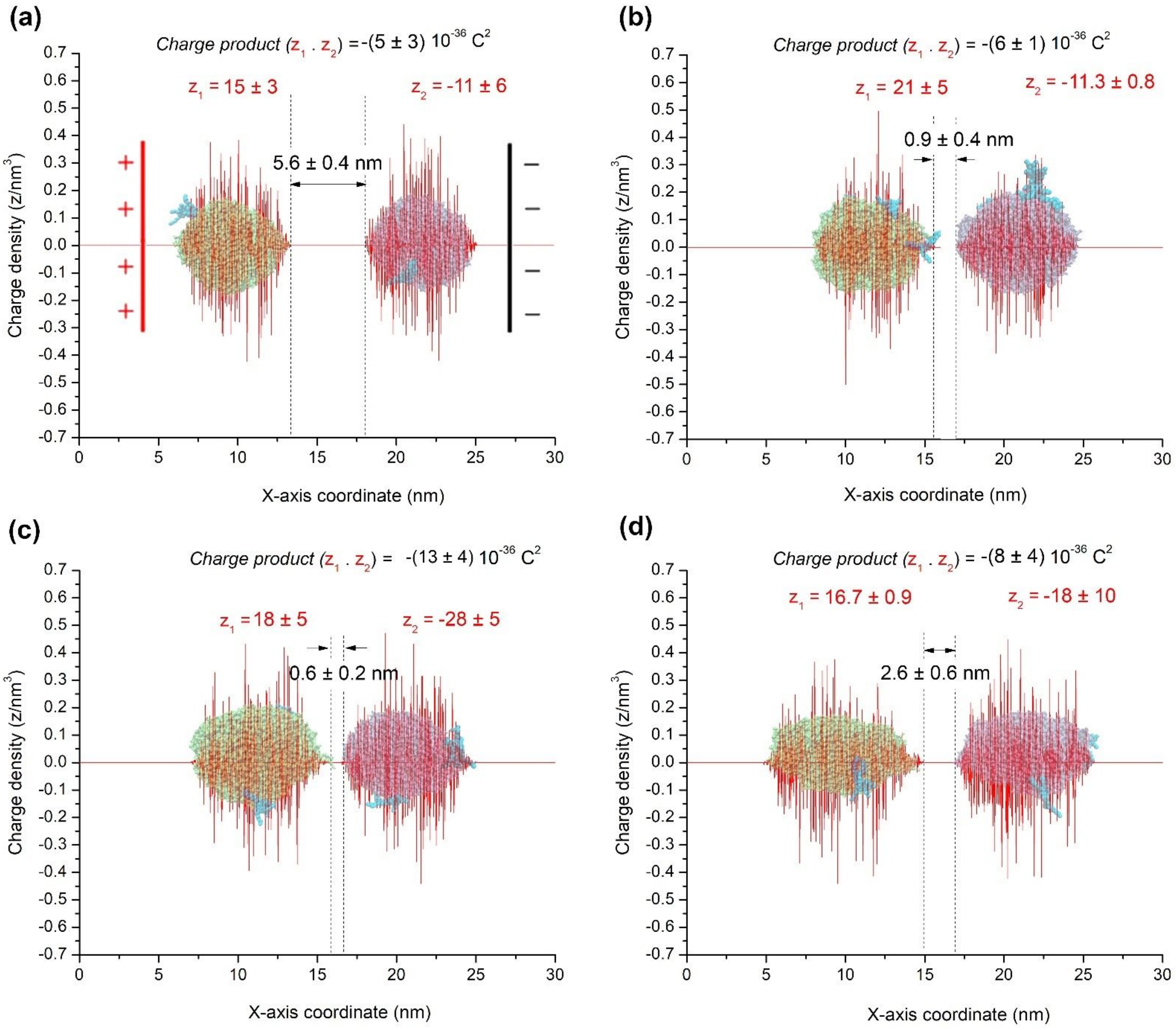

Figure S6. Distribution of charge density ($\rho(x)$ in solid red lines) of two coalescing water droplets with 3 asphaltene molecules under DC electrical field (E): (**a**) 0.3 V/nm, (**b**) 0.4 V/nm, (**c**) 0.5 V/nm, (**d**) 0.6 V/nm applied along the x-axis of simulation box. The asphaltene molecules on water/oil interface are represented in van deer Waals notation with cyan color. The electrical charge of each droplet (z_1 and z_2) before forming a liquid-bridge in these emulsions was estimated by **Eq. 8** in the text. The distance (d) found by NPT simulations between leading edges of droplets is represented between arrows. The electrical charge product ($z_1 \,.\, z_2$ in Coulomb units (C) is shown for each system. The geometric mean electrical charge at the interface was estimated by $\sqrt{|z_1 \,.\, z_2|}$, which ranges from 2.2 to 3.6 x 10^{-18} C and agrees with computed saturation charge ($q_{s,x}$, 4.3 - 8.6 x 10^{-18} C) of a conductive particle of radius r_x with an electrode.[8] The sodium cations contained within the droplets and n-hexane molecules in the W/O systems are not depicted for clarity reasons.

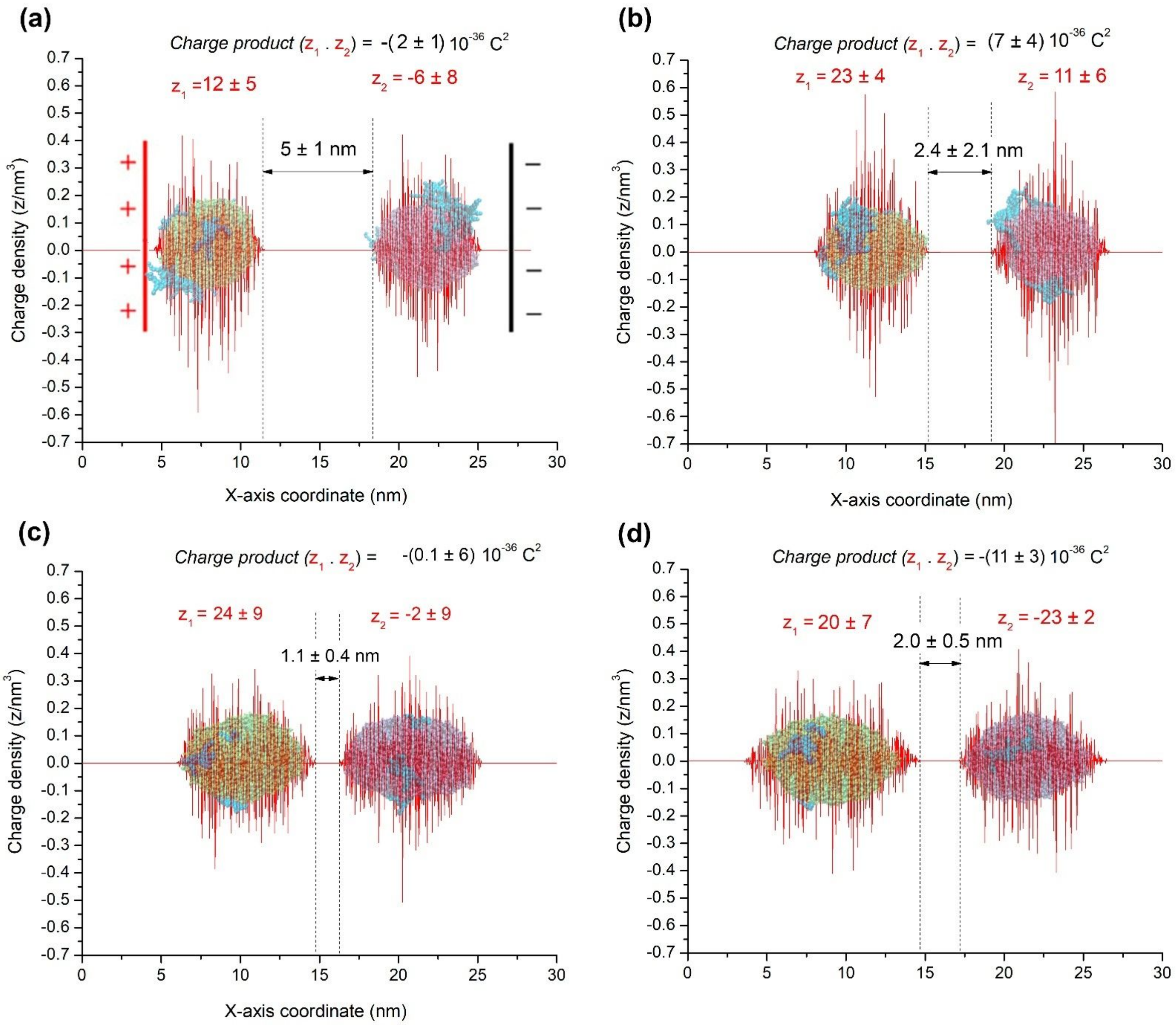

Figure S7. Distribution of charge density ($\rho(x)$ in solid red lines) of two coalescing water droplets with 20 asphaltene molecules under DC electrical field (E): (**a**) 0.3 V/nm, (**b**) 0.4 V/nm, (**c**) 0.5 V/nm, (**d**) 0.6 V/nm applied along the x-axis of simulation box. The asphaltene molecules on water/oil interface are represented in van deer Waals notation with cyan color. The electrical charge of each droplet (z_1 and z_2) before forming a liquid-bridge in these emulsions was estimated by **Eq. 8** in the text. The distance found by NPT simulations between leading edges of droplets (d) is represented between arrows. The electrical charge product ($z_1 . \ z_2$ in Coulomb units (C) is shown for each system. The geometric mean electrical charge at the interface was estimated by $\sqrt{|z_1 . \ z_2 \,|}$, which ranges from 0.3 to 3.3 x 10^{-18} C and agrees with computed saturation charge ($q_{s,x}$, 4.3 - 8.6 x 10^{-18} C) of a conductive particle of radius r_x with an electrode.[8] The sodium cations contained within the droplets and n-hexane molecules in the W/O systems are not depicted for clarity reasons.

Note S2. Estimation of surface/interfacial tension (γ) at the interface between leading edges of water droplets

Approximate values of γ for the anionic asphaltenes in our W/O systems can be estimated by Langmuir equation of state:[16]

$$\gamma(\Gamma) = \gamma_0 + kT\Gamma_\infty \ln\left(1 - \Gamma/\Gamma_\infty\right) \quad \textbf{(S21)}$$

where γ_0 is the clean surface interfacial tension of hexane/water (50.7 mN/m) [6] and Γ_∞ is maximum interfacial coverage, ~3.2 molecule/nm^2.[9,10] To estimate the interfacial coverage (Γ), the following equation was used:

$$\Gamma = \frac{N_{\text{asph}}^{\text{inter}}}{\mathrm{A_s}} = \frac{N_{\text{asph}}^{\text{inter}}}{\mathrm{A_s^1 + A_s^2}} \quad \textbf{(S22)}$$

Here, $N_{\text{asph}}^{\text{inter}}$ (**Table 3** in the text) is the total amount of asphaltene molecules accumulated at the interface between the leading edges of colliding water droplets. Since $N_{\text{asph}}^{\text{inter}}$ considers the asphaltene number on two different surfaces of water droplets: $\mathrm{A_s^1}$ for droplet 1 and $\mathrm{A_s^2}$, then $\mathrm{A_s = A_s^1 + A_s^2}$. The main difficulty for an accurate estimation of γ using **Eq. S21** and **S22** is precisely the determination of $\mathrm{A_s}$ (interfacial surface occupied by anionic asphaltenes on between the leading edges of water droplets). To perform the calculation of $\mathrm{A_s}$, we considered that $\mathrm{A_s^1}$ is for example:

$\mathrm{A_s^1} = 2\pi * r * h$ (surface area of cap sphere of water droplet 1).[11] r is the radius (= 3 nm) of water droplet and h ($= 6 * \mathrm{D_1}$, see **Table S2**) is the deformed distance of water droplet 1 by applying the electric field. The same methodology was utilized for the calculation of $\mathrm{A_s^2}$.

Then:

$$\mathrm{A_s} \cong 72\pi * (\mathrm{D_1 + D_2}) \quad \textbf{(S23)}$$

The values of $\mathrm{A_s}$ were in the range of 19-118 nm^2 (**Table S2**), which should be roughly from 1 to 4 nm^2.[13 12] Therefore, $\mathrm{A_s}$ was scaled by 0.1 in **Eq. S23**

Substituting values of $N_{\text{asph}}^{\text{inter}}$ (**Table 3** in the text), $\mathrm{D_1}$ and $\mathrm{D_2}$ of water droplets (**Table S2**) into **Eq. S22-S23**, we get values of γ around 44.6 – 50.6 mN/m, as shown in **Table 3** in the text. Note that the values of γ are close to the water-in-hexane (50.7 mN/m) because the relative coverage (Γ/Γ_∞) of asphaltenes between the leading edges is very low: 0 - 0.011 and 0.07 - 0.7 for system with $N = 3$ and $N = 20$, respectively. As theoretically expected, the increase in γ is mainly due to the increase in $\mathrm{A_s}$ with increasing E.

On the other hand, the error in γ can be estimated by differentiating the Eq. S21, which renders:

$$d\gamma = kT\Gamma_\infty \frac{d(1-\Gamma/\Gamma_\infty)}{(1-\Gamma/\Gamma_\infty)} \quad \textbf{(S24)}$$

Substituting k, Γ_∞ and $T = 300\ K$ in convenient units to provide mN/m, we have that:

$$\Delta\gamma = 4.14 \frac{\Delta\Gamma}{(1-\Gamma/3.2)} \quad \textbf{(S25)}$$

Since $\Gamma = \frac{N_{\text{asph}}^{\text{inter}}}{\mathrm{A_s}}$, both the numerator and denominator have errors (see **Table 3** in the text and last column in **Table S2**). Therefore, we can then evaluate the error in Γ applying errors propagation theory, that is:

$$\Delta\Gamma = \left(\frac{\Delta N_{\text{asph}}^{\text{inter}}}{N_{\text{asph}}^{\text{inter}}} + \frac{\Delta \mathrm{A_s}}{\mathrm{A_s}}\right)\frac{N_{\text{asph}}^{\text{inter}}}{\mathrm{A_s}} \quad \textbf{(S26)}$$

Table S5. Percent (Q in %) [a)] of asphaltenes on the adjacent interface per each droplet at different electric field strength (E in V/nm) and number of asphaltenes per droplet (N) in the W/O emulsions

	r_1 [b)]	Q_1	r_2	Q_2	Q_{total} [c)]	N_{asph}^{inter} [d)]
E (N)	**Droplet 1**		**Droplet 2**			
0.3 (3)	1.65 ± 0.03	0.0 ± 0.0	1.86 ± 0.06	4 ± 2	4 ± 2	0.12 ± 0.06
0.4 (3)	2.0 ± 0.2	14 ± 1	2.0 ± 0.1	1.1 ± 0.3	15 ± 1	0.45 ± 0.03
0.5 (3)	2.40 ± 0.06	13 ± 5	2.3 ± 0.1	0.3 ± 0.1	13 ± 5	0.4 ± 0.1
0.6 (3)	2.9 ± 0.1	13 ± 3	2.8 ± 0.2	0.0 ± 0.0	13 ± 3	0.39 ± 0.09
0.3 (20)	1.7 ± 0.1	3 ± 2	1.9 ± 0.1	14 ± 4	17 ± 4	3.4 ± 0.8
0.4 (20)	2.01 ± 0.03	6 ± 4	2.3 ± 0.1	12 ± 2	18 ± 4	3.6 ± 0.8
0.5 (20)	2.88 ± 0.06	5 ± 1	2.5 ± 0.2	19 ± 2	24 ± 2	4.8 ± 0.4
0.6 (20)	3.3 ± 0.2	4.2 ± 0.7	2.8 ± 0.2	8 ± 3	12 ± 3	2.4 ± 0.6

a) Q_1 and Q_2 is the ratio of asphaltene number at the interface to total number for water droplet 1 and 2, respectively

b) $r_1(= 1.5 + 6 * \mathrm{D}_1)$ is the slice thickness used to calculate the integration limits (see **Eq. 12** in the text). D_1 is the deformation ratio of the droplet 1 (**Table S2**)

c) Q_{total} is the sum of Q_1 and Q_2 which represents the total percent of asphaltenes accumulated on the adjacent interface between water droplets

d) N_{asph}^{inter} ($=N*Q_{total}/100$) is the number of asphaltenes accumulated on the adjacent interface between water droplets.

Table S6. Distances (δ in nm) of sodium cations from the interior to the surface of the water droplets at different electric field strength (E) and number of asphaltenes per droplet (N) in W/O emulsions

E (V/nm)	δ_1/ δ_2 [a]	
	$N = 3$	$N = 20$
0.3	$0.9 \pm 0.1/3.4 \pm 0.7$	$0.5 \pm 0.4/1.6 \pm 0.9$
0.4	$0.6 \pm 0.1/2.0 \pm 0.4$	$0.70 \pm 0.03/1.4 \pm 0.2$
0.5	$1.0 \pm 0.2/3.0 \pm 0.1$	$1.1 \pm 0.1/1.0 \pm 0.3$
0.6	$2.7 \pm 0.1/2.5 \pm 1.3$	$0.8 \pm 0.1/1.2 \pm 0.1$

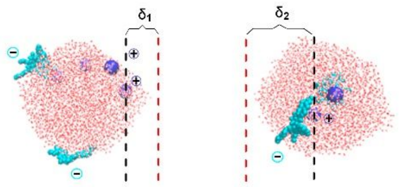

a) δ_1 and δ_2 stand for the minimal distances at which is found sodium cations from the interior to the surface in the left (droplet 1) and right droplet (droplet 2), respectively. The distances were calculated from mass density distribution of sodium ions in each droplet before forming the liquid bridge (**Figure 13b** and **13d** in the text)

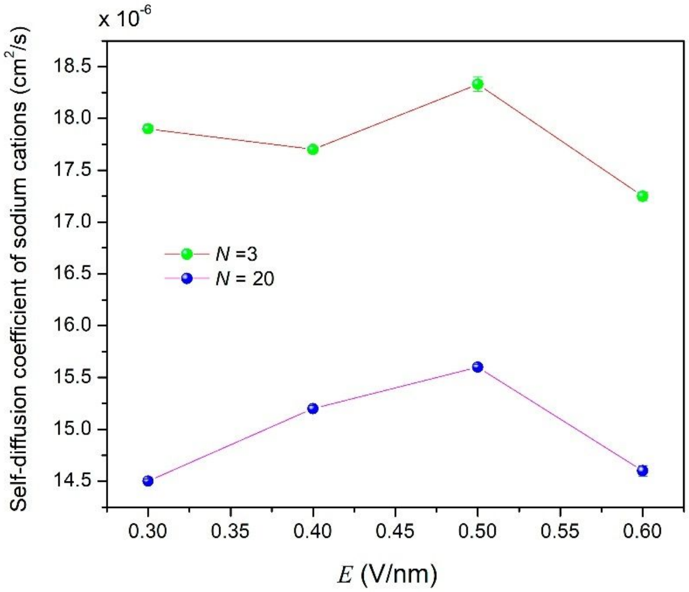

Figure S8. Self-diffusion coefficient of sodium cations (D_{Na}) within water droplets (D_{Na}) at different electric field strengths (E): 0.3-0.6 V/nm for $N = 3$ and $N = 20$. Plotted data is assembled in **Table S7**

Table S7. Conductivity (σ) [a] and self-diffusion coefficient (D) [b] of asphaltenes ($\mathrm{SJ15}$), sodium cations (Na) and water molecules in the droplets at different electric field strength (E in V/nm) and number of asphaltenes per droplet (N) in W/O emulsions

	D_{SJ15} x 10^{-6} cm^2/s	σ_{SJ15} S/m	D_{Na} x 10^{-6} cm^2/s	σ_{Na} S/m	D_{water} x 10^{-5} cm^2/s	Droplet conductivity ($\sigma_{\mathrm{SJ15}} + \sigma_{\mathrm{Na}}$) S/m
E (N)						
0.3 (0)	--	--	--	--	3.2 ± 0.01	--
0.4 (0)	--	--	--	--	3.30 ± 0.01	--
0.5 (0)	--	--	--	--	3.25 ± 0.01	--
0.6 (0)	--	--	--	--	3.1 ± 0.01	--
0.3 (3)	9.25 ± 0.02	0.15	17.9 ± 0.03	0.293	3.22 ± 0.01	0.443
0.4 (3)	12 ± 0.08	0.197	17.7 ± 0.03	0.29	3.33 ± 0.01	0.487
0.5 (3)	11.03 ± 0.08	0.18	18.33 ± 0.07	0.30	3.28 ± 0.01	0.48
0.6 (3)	10.9 ± 0.09	0.179	17.25 ± 0.04	0.282	3.22 ± 0.01	0.461
0.3 (20)	8.92 ± 0.007	0.976	14.5 ± 0.02	1.587	2.98 ± 0.01	2.563
0.4 (20)	9.0 ± 0.04	0.985	15.2 ± 0.03	1.663	3.05 ± 0.01	2.648
0.5 (20)	8.08 ± 0.03	0.88	15.6 ± 0.03	1.707	3.04 ± 0.01	2.587
0.6 (20)	7.8 ± 0.02	0.853	14.6 ± 0.05	1.598	3.01 ± 0.01	2.451

a) The ionic conductivity for each specie was estimated from estimated D [13,14] (**Section 3.4.6** in the text)

b) The diffusion coefficient (D) of species was calculated by Einstein's relationship using NVT ensemble [15,16] (**Section 3.4.6** in the text)

Table S8. Dipole moment (in Debye) along X-direction of simulation box at different electric field strength (E) and number of asphaltenes per water droplet (N) in W/O emulsions.

E (V/nm)	N		
	0	3	20
0.3	652 ± 7	468 ± 168	86 ± 23
0.4	897 ± 17	1341 ± 299	129 ± 88
0.5	1623 ± 3	1801 ± 144	930 ± 450
0.6	1780 ± 428	3429± 739	1952 ± 75

Note S3. Estimation of dipole forces by scaling analysis

Using the scaled equations for droplet forces in electro-coalescence,[17] it is possible to determine the magnitude of dipole forces. The dipolar attraction force (F_a) can be estimated for a droplet distance equal to one particle diameter as follows:

$$\frac{F_a}{F_g} \sim 15 \qquad \textbf{(S31)}$$

Where F_a and F_g are the dipolar attraction and gravity forces, respectively. F_g can be calculated as:

$$F_g = \boldsymbol{m}(\rho_w - \rho_o)\boldsymbol{g} \qquad \textbf{(S32)}$$

Where $\boldsymbol{m}$ is the droplet mass, $\boldsymbol{g}$ is the gravity field, ρ is the density, and the subscripts *w* and *o* refer to water and oil, respectively. In our system, at room condition, the ρ_o (hexane density) and ρ_w is equal to 660 kg/m^3 and 998 kg/m^3, respectively. Taking into account 3770 water molecules in the droplet, we have a value of $\boldsymbol{m}$ equal to 1.13 10^{-22} kg. Therefore from **Eq. S32** and **S31** we have $F_a =$ 5.6 10^{-18} N. This value is approximately 6 orders of magnitude lower than attraction dipole forces (F_{dip}) between water droplets obtained by MD simulations (**Figure 18** in the text). That is, $F_{\mathrm{dip}} > F_a$ due to the stronger electric field applied to nanometric size droplets compared to experiments.

Note S4. Derivation of the error formulation for dipoles forces (F_{dip}) according to errors propagation theory

According to the **Eq. 9** in the text, the electrostatic force of dipoles is defined as follows:

$$F_{\text{dip}} = \frac{-12\pi\omega^2\ \epsilon_m E^2 r_1^3 r_2^3}{\mathrm{S}^4}(3K-1) \quad \textbf{(S41)}$$

The error in F_{dip} (ΔF_{dip}) is calculated on the basis of total differential (dF_{dip}). Thus, by using the total differential, we can estimate the approximate change in the function's value due to these input errors, which essentially provides an approximation of the error in the function's output.[18]

Since F_{dip} is a function of errors in the variables K and S, we have that:

$$dF_{\text{dip}} = \left(\frac{\partial F_{\text{dip}}}{\partial \mathrm{S}}\right)_{\mathrm{K}} d\mathrm{S} + \left(\frac{\partial F_{\text{dip}}}{\partial \mathrm{K}}\right)_{\mathrm{S}} \frac{\partial F_{\text{dip}}}{\partial \mathrm{K}} d\mathrm{K} \quad \textbf{(S42)}$$

Here, it is considered that E and water droplets radius (r_1 and r_2 equal to $a = 3$ nm) do not have errors, and they are defined in the simulation experiments.

$$\left(\frac{\partial F_{\text{dip}}}{\partial \mathrm{S}}\right)_{\mathrm{K}} = \frac{48\pi\omega^2\ \epsilon_m E^2 r_1^3 r_2^3}{\mathrm{S}^5}(3K-1);\ \left(\frac{\partial F_{\text{dip}}}{\partial \mathrm{K}}\right)_{\mathrm{S}} = \frac{-36\pi\omega^2\ \epsilon_m E^2 r_1^3 r_2^3}{\mathrm{S}^4}$$

By inserting these partial derivates into **Eq. S42** and making some algebraic operations, we get:

$$dF_{\text{dip}} = \frac{-12\pi\omega^2\ \epsilon_m E^2 a^6}{\mathrm{S}^4}\left\{3\ d\mathrm{K} - \frac{4}{\mathrm{S}}(3\mathrm{K}-1)d\mathrm{S}\right\} \quad \textbf{(S43)}$$

Thus, as an approximation in error propagation theory,[19] we have that:

$$\Delta F_{\text{dip}} = \frac{-12\pi\omega^2\ \epsilon_m E^2 a^6}{\mathrm{S}^4}\left\{3\Delta\mathrm{K} - \frac{4}{\mathrm{S}}(3\mathrm{K}-1)\Delta\mathrm{S}\right\} \quad \textbf{(S44)}$$

The reported values of K (**Table S3**) and S (**Table S4**), together with their errors (ΔK and ΔS) allows to determine ΔF_{dip} at different electric field strength (E).

Table S9. Calculated values of dipole forces (in Newton) [a] between water droplets before creating the liquid bridge at different electric field strength (E) and number of asphaltenes per droplet (N) in W/O emulsions. Values are illustrated in **Figure 18** in the text

E (V/nm)	N		
	0	3	20
0.3	$-(5.1 \pm 0.3)\ 10^{-13}$	$-(9.0 \pm 0.1)\ 10^{-13}$	$-(4.1 \pm 0.3)\ 10^{-13}$
0.4	$-(3.0 \pm 1.6)\ 10^{-12}$	$-(6.6 \pm 0.1)\ 10^{-12}$	$-(3.0 \pm 0.7)\ 10^{-12}$
0.5	$-(4.1 \pm 0.1)\ 10^{-12}$	$-(7.1 \pm 0.3)\ 10^{-12}$	$-(4.6 \pm 0.7)\ 10^{-12}$
0.6	$-(2.0 \pm 0.5)\ 10^{-12}$	$-(1.9 \pm 0.3)\ 10^{-12}$	$-(1.5 \pm 0.04)\ 10^{-12}$

a) The calculation was performed by **Eq. 9** in the text considering S estimated from atoms trajectory motion.

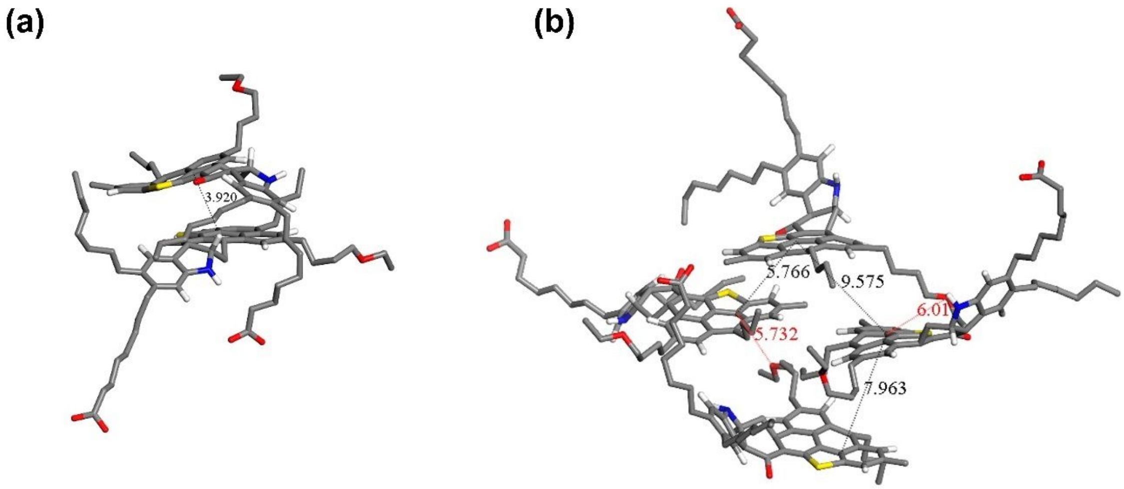

Figure S9. Structure of the multimers [dimer (a), tetramer (b)] found during droplet-droplet coalescence in the emulsion system with 20 anionic asphaltene molecules applying an electric field (E) equal to 0.6 V/nm (see **Video S4**). These structures are formed before connection between colliding droplets by the liquid bridge (LB). Once the droplets touch each other by LB, WCC is formed with extensive disaggregation of the multimers. In figures are shown the distances between aromatic rings centroids in face-to-face π-π stacking (*in black*) and ether oxygen of the aliphatic chains with the conjugated aromatic rings due to the n→π interaction (*in red*).[19] At high E, it is possible that in tetramers (**Figure S9b**) the n→π interaction acts synergistically with face-to-face π-π stacking interaction even though the interaction distance between ring centroids is very large (> 7 Å).

References

1. Wang, Z.; Dong, K.; Tian, L.; Wang, J.; Tu, J. Numerical study on coalescence behavior of suspended drop pair in viscous liquid under uniform electric field. *AIP Advances* **2018**, *8*, 085215.

2. Torza, S.; Cox, R. G.; Mason, S. G. Electrohydrodynamic deformation and bursts of liquid drops. *Philos. Trans. R. Soc. A* **1971**, *269*, 295-319.

3. Mhatre, A.; Deshmukh, S.; Thaokar, R. M. Electrocoalescence of a drop pair. Physics of Fluids 2015, 27, 092106.

4. Giljarhus, K. E. T.; Munkejord, S. T. Numerical investigation of electrostatically enhanced coalescence of two drop in a flow field, in IEEE International Conference on Dielectric Liquids (IEEE, 2011), pp. 1–4

5. Visser, J. On Hamker constants: A comparison between Hamaker constants and Lifshitz - Van der Waals constants. *Advan. in Colloid and Interface Sci.* **1972**, *3*, 331-363.

6. Saien, J.; Rezvani, A.; Asadabadi, S. Interfacial Tension of the n-Hexane–Water System under the Influence of Magnetite Nanoparticles and Sodium Dodecyl Sulfate Assembly at Different Temperatures. *Journal of Chemical & Engineering Data* **2014**, *59*, 1835-1842.

7. Kwan, V.; Consta, S. Molecular Characterization of the Surface Excess Charge Layer in Droplets. *J. Am. Soc. Mass Spectrom.* **2021**, *32*, 33-45.

8. Sadek, S. E.; Hendricks, C. D. Electrical coalescence of water droplets in low-conductivity oils. *Ind. Eng. Chem. Fundamen.* **1974**, *13*, 139-142.

9. Rane, J. P.; Pauchard, V.; Couzis, A.; Banerjee, S. Interfacial Rheology of Asphaltenes at Oil−Water Interfaces and Interpretation of the Equation of State. *Langmuir* **2013**, *29*, 4750−4759.

10. Pauchard, V. O.; Rane, J. P.; Banerjee, S. Asphaltene-laden interfaces form soft glassy layers in contraction experiments: a mechanism for coalescence blocking. *Langmuir* **2014**, *30*, 2795-12803.

11. AmBRBit. https://www.ambrbit.com/TrigoCalc/Sphere/Cap/SphereCap.htm. Accessed 06/2025

12. Rogel, E.; León, O.; Torres, G.; Espidel, J. Aggregation of asphaltenes in organic solvents using surface tension measurements. *Fuel* **2000**, *79*, 1389-1394.

13. Zang, Y. -J. Entropy and ionic conductivity. *Physica A: Statistiscal Mechanics and its applications* **2012**, *391*, 4470-4475.

14. Park, M.; Zhang, X.; Chung, M.; Less, G. B.; Sastry, A. M. A review of conduction phenomena in Li-ion batteries. *Journal of Power Sources* **2010**, *195*, 7904-7929.

15. Baba, H.; Urano, R.; Nagai, T.; Okazaki, S. Prediction of self-diffusion coefficients of chemically diverse pure liquids by all-atom molecular dynamics simulations. *Journal of computational Chemistry* **2022**, *43*, 1892-1900.

16. Ghaffari, A.; Rahbar-Kelishami, A. MD simulation and evaluation of the self-diffusion coefficients in aqueous NaCl solutions at different temperatures and concentrations. *Journal of Molecular Liquids* **2013**, *187*, 238-245.

17. Esteves, B. F.; Castor Jr., C. A.; Pinto, J. C.; Nele, M. Scaling analysis of droplet forces in electrocoalescence: effect of frequency and waveform on dehydration efficiency. *Brazilian Journal of Chemical Engineering* **2024**, *41*, 1285-1304.

18. Ku, H. H. Notes on the use of propagation of errors formulas*. Journal of Research of the National Bureaus of Standards - C. Engineering and Instrumentation* **1966**, *70C*, 263-273.

19. Peksoz, A.; Almaz, E.; Yalciner, A. The characterization of asphaltene behavior in some aromatic solvents by dynamic nuclear polarization technique. *Journal of Petroleum Science and Engineering* **2010**, *75*, 58-65.
